# Anakinra restores cellular proteostasis by coupling mitochondrial redox balance to autophagy

**DOI:** 10.1172/JCI144983

**Published:** 2022-01-18

**Authors:** Frank L. van de Veerdonk, Giorgia Renga, Marilena Pariano, Marina M. Bellet, Giuseppe Servillo, Francesca Fallarino, Antonella De Luca, Rossana G. Iannitti, Danilo Piobbico, Marco Gargaro, Giorgia Manni, Fiorella D’Onofrio, Claudia Stincardini, Luigi Sforna, Monica Borghi, Marilena Castelli, Stefania Pieroni, Vasileios Oikonomou, Valeria R. Villella, Matteo Puccetti, Stefano Giovagnoli, Roberta Galarini, Carolina Barola, Luigi Maiuri, Maria Agnese Della Fazia, Barbara Cellini, Vincenzo Nicola Talesa, Charles A. Dinarello, Claudio Costantini, Luigina Romani

**Affiliations:** 1 Department of Medicine, Radboud University Medical Center, Nijmegen, Netherlands.; 2 Department of Medicine and Surgery, University of Perugia, Perugia, Italy.; 3 European Institute for Research in Cystic Fibrosis, Division of Genetics and Cell Biology, San Raffaele Scientific Institute, Milan, Italy.; 4 Department of Pharmaceutical Sciences, University of Perugia, Perugia, Italy.; 5 Istituto Zooprofilattico Sperimentale dell’Umbria e delle Marche “Togo Rosati,” Perugia, Italy.; 6 Department of Health Sciences, University of Piemonte Orientale, Novara, Italy.; 7 Department of Medicine, University of Colorado, Aurora, Colorado, USA.

**Keywords:** Infectious disease, Inflammation, Autophagy, Fungal infections

## Abstract

Autophagy selectively degrades aggregation-prone misfolded proteins caused by defective cellular proteostasis. However, the complexity of autophagy may prevent the full appreciation of how its modulation could be used as a therapeutic strategy in disease management. Here, we define a molecular pathway through which recombinant IL-1 receptor antagonist (IL-1Ra, anakinra) affects cellular proteostasis independently from the IL-1 receptor (IL-1R1). Anakinra promoted H_2_O_2_-driven autophagy through a xenobiotic sensing pathway involving the aryl hydrocarbon receptor that, activated through the indoleamine 2,3-dioxygenase 1-kynurenine pathway, transcriptionally activated NADPH oxidase 4 independent of the IL-1R1. By coupling the mitochondrial redox balance to autophagy, anakinra improved the dysregulated proteostasis network in murine and human cystic fibrosis. We anticipate that anakinra may represent a therapeutic option in addition to its IL-1R1–dependent antiinflammatory properties by acting at the intersection of mitochondrial oxidative stress and autophagy with the capacity to restore conditions in which defective proteostasis leads to human disease.

## Graphical abstract

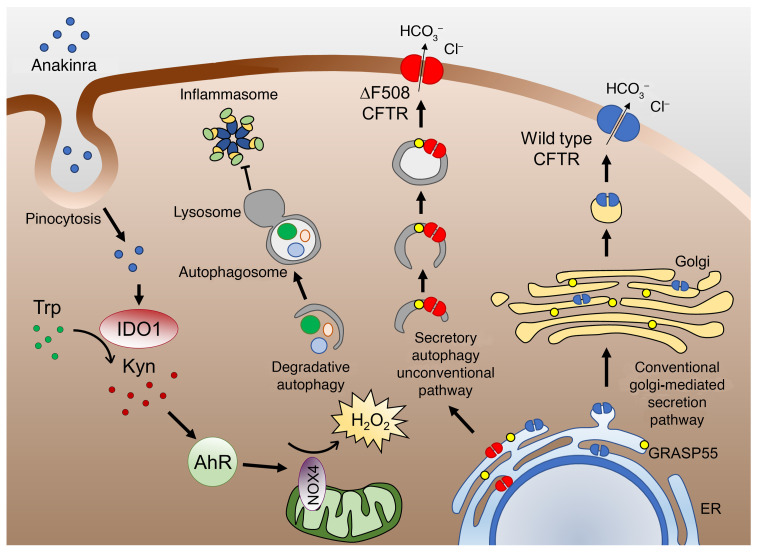

## Introduction

The lung is equipped with a robust proteostasis network for handling protein folding, misfolding, unfolding, and degradation in response to mechanical and environmental stress (1). Consistent with the emerging importance of the autophagy pathway in multiple human pulmonary diseases (2), autophagy is required in the maintenance of cellular proteostasis (3). Autophagy selectively degrades aggregation-prone misfolded proteins, such as those involved in the pathogenesis of certain neurodegenerative and lung diseases and aging (4). In certain types of lung diseases, autophagy may have a protective role to allow handling of misfolded proteins and prevent inflammatory damage. In cystic fibrosis (CF), an autosomal recessive disorder caused by mutations in the gene encoding for the CF transmembrane conductance regulator (CFTR; ref. 5), disruption of autophagy could promote pathogenesis of the disease (6). Defective autophagy has indeed been observed in cells expressing the most common mutant variant p.Phe508del-CFTR and accumulating polyubiquitinated proteins and aggresome-like structures. Given that functional autophagy and proteostasis have been shown to promote trafficking, plasma membrane targeting, and stability of defective CFTR (7, 8), strategies aiming at restoring autophagy have been proposed as a therapeutic approach in CF (7, 9). Cysteamine and an endogenously occurring nitric oxide donor are well-known examples of proteostasis and autophagy regulators endowed with therapeutic efficacy in preclinical CF (6). However, the complexity of autophagy may prevent the full appreciation of how its modulation could be used as a therapeutic strategy in disease management. This complexity necessitates preclinical studies aimed at defining the molecular pathways of autophagy and their relevance to disease pathogenesis.

Over the past 30 years, IL-1–mediated inflammation has been established in a broad spectrum of diseases. Quenching IL-1–mediated inflammation with anakinra has provided clinically evident benefits associated with an unparalleled record of safety. By mimicking the naturally occurring IL-1 receptor antagonist (IL-1Ra), anakinra — which differs from IL-1Ra by the presence of an extra methionine in the amino terminus and the absence of glycosylation — exerts its effects by inhibiting the binding of IL-1β and IL-1α to IL-1 receptor (IL-1R1) (10). Anakinra is presently in clinical trials to treat cancer, and off-label use of anakinra far exceeds its approved indications (10, 11). This raises the intriguing hypothesis that anakinra may have effects beyond its receptor-inhibiting activity. Besides a secreted protein, 3 intracellular, un-secreted isoforms of IL-1Ra have been described in humans and in mouse tissues (12). Whereas extracellular IL-1Ra inhibits IL-1 activity by binding to IL-1R1, intracellular IL-1Ra1 was shown to exhibit antiinflammatory properties (13), thus suggesting that anakinra may have a receptor-independent activity. In this regard, the facts that (a) anakinra has shown a superior activity compared with IL-1β neutralizing antibody on murine lung infection and pathology (14), (b) cellular uptake of exogenous anakinra has been described (15), and (c) surface-bound IL-1Ra does not undergo receptor-mediated internalization (16) all suggest that anakinra may exert receptor-independent intracellular activities.

In the present study, we have characterized the process of autophagy induced by anakinra, its dependency on receptor activity, and its possible role in lung proteostasis. By using preclinical models of CF in vitro and in vivo, we have discovered that anakinra promotes IL-1R1–independent autophagy through the aryl hydrocarbon receptor that, activated through the indoleamine 2,3-dioxygenase 1-kynurenine pathway, leads to NOX4-dependent mitochondrial H_2_O_2_ production and positively regulates cellular proteostasis in mice and human cells with the p.Phe508del-CFTR mutation.

## Results

### Anakinra induces IL-1R1–independent autophagy.

**Figure 1:**
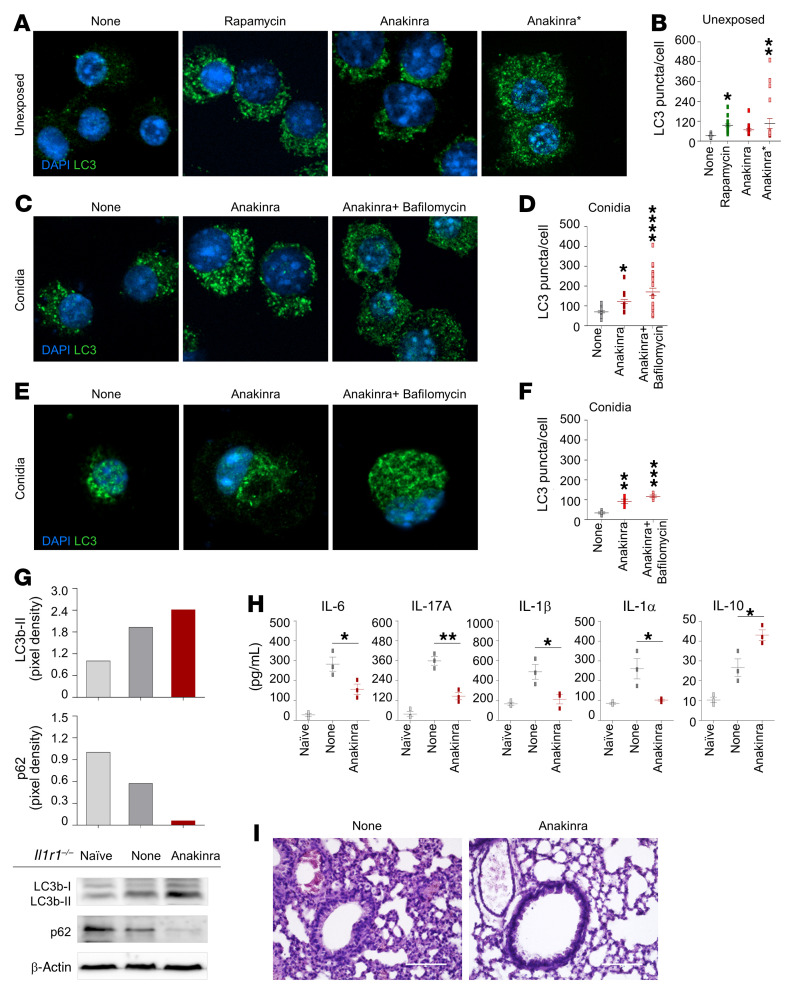
Anakinra induces autophagy and limits inflammation in the absence of IL-1R1. (**A**–**D**) LC3 staining of RAW 264.7 cells treated with 50 μM rapamycin, 10 μg/mL anakinra, or a truncated form (Anakinra*) (**A **and** B**); exposed to *A*. *fumigatus* conidia and treated with anakinra with and without 100 nM bafilomycin for 4 hours (**C** and** D**). (**E** and **F**) LC3 staining of alveolar macrophages purified from *Il1r1^–/–^* naive mice exposed to *A*. *fumigatus* conidia and treated with anakinra and/or bafilomycin as above. (**B**,** D**, and** F**) Mean percentage of LC3 puncta/cells (*n =* 4–22). Data represent the mean ± SEM of 1 representative out of 3 (**A**–**D**) or 2 (**E **and** F**) independent experiments. DAPI was used to detect nuclei. (**G**–**I**) *Il1r1^–/–^* mice were infected (i.n.) with live *A*. *fumigatus* conidia, treated with 10 mg/kg anakinra (i.p.) as described in Methods, and assessed for immunoblotting of LC3b and p62 (**G**), cytokine production (ELISA) in lung homogenates (**H**), and lung histology [periodic acid-Schiff (PAS) staining] (**I**) at 7 days after infection. Scale bar: 200 μm. (**G** and** I**) A representative experiment is shown; (**H**) data represent 3 independent experiments. Each independent in vivo experiment includes 6–8 mice per group pooled before analysis. *P < 0.05, **P < 0.01, ***P < 0.001, ****P < 0.0001, treated versus untreated (None) cells. One-way ANOVA, Bonferroni post hoc test.

We have already shown that anakinra dose-dependently decreased inflammasome activity in *Aspergillus fumigatus* infection via autophagy (14, 17). We found here that anakinra promoted autophagy in the relative absence of IL-1R1 both in vitro and in vivo. In RAW 264.7 cells in which defective NLRP3 activity (18) allows uncoupling of the intracellular effects of anakinra from the inflammasome (Figure 1, A–D) and in purified *Il1r1*^–/–^ lung macrophages (Figure 1, E and F), anakinra increased the number of LC3 punctae in cells either unexposed or after exposure to *Aspergillus* conidia. LC3 expression was increased in the presence of either bafilomycin (Figure 1, C and D) or chloroquine (Supplemental Figure 1; supplemental material available online with this article; https://doi.org/10.1172/JCI144983DS1), suggesting that anakinra induces an increase in autophagosomal turnover. Autophagy was still observed with a truncated form of anakinra obtained by limited proteolysis of the N-terminus, likely affecting the receptor binding site of the molecule (ref. 19, Figure 1, A and B, and Supplemental Figure 2). In vivo, treatment of *Aspergillus*-infected *Il1r1*^–/–^ mice with anakinra increased the ratio of LC3-II/I and decreased p62, a ubiquitin-binding protein that is selectively degraded by autophagy (Figure 1G); inhibited IL-6, IL-17A, IL-1β, and IL-1α production; increased IL-10 (Figure 1H); and ameliorated lung inflammation (Figure 1I). Similar results were obtained in *Pseudomonas aeruginosa*–infected *Il1r1*^–/–^ mice (Supplemental Figure 3). These findings suggest that anakinra promotes autophagy and shows antiinflammatory activity beyond its competitive inhibition of IL-1 binding to IL-1R1.

### Anakinra affects mitochondrial redox balance via NOX4.

**Figure 2:**
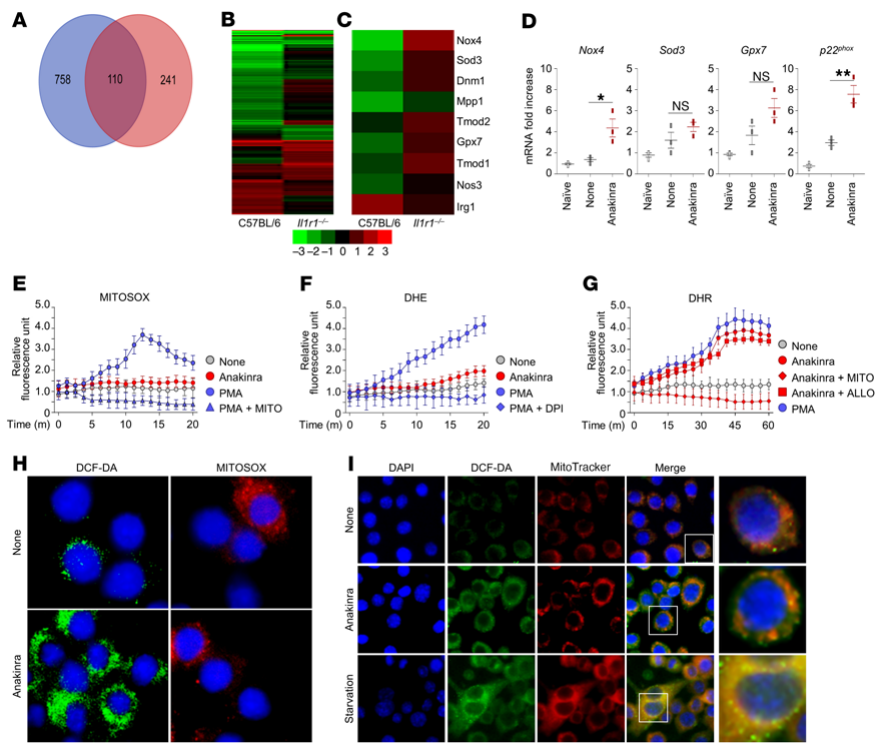
Anakinra affects mitochondrial redox balance in the absence of IL-1R1. (**A**) Genes differentially expressed in response to anakinra in purified alveolar macrophages from C57BL/6 and *Il1r1^–/–^* mice stimulated in vitro with 10 μg/mL of anakinra for 4 hours. (**B** and **C**) Hierarchical clustering (fold change > 2, *P* value < 0.05, FDR < 0.05) by RNA-Seq of all genes (**B**) and genes involved in oxidative stress (**C**). (**D**) RT-PCR on total lung cells from *Il1r1^–/–^*mice at 7 days after *A*. *fumigatus* i.n. infection. *P < 0.05 and **P < 0.01, anakinra-treated versus untreated (None) mice. NS, not statistically significant. One-way ANOVA, Bonferroni post hoc test. Data are the mean ± SEM of 1 representative out of 3 independent experiments. (**E–G**) RAW 264.7 cells were exposed to 10 ng/mL PMA or 10 μg/mL anakinra for 4 hours at 37°C in the presence of diphenylene iodonium (DPI), allopurinol (ALLO), or MitoTEMPO (MITO) with the addition of MitoSOX red (**E**), DHE (**F**), and DHR (**G**) for mitochondrial superoxide, NADPH-dependent superoxide, and mitochondrial hydrogen peroxide assessment, respectively. Fluorescence was measured at 510 excitation and 580 emission. The results presented for all fluorimetric measurements are the mean ± SEM of 1 representative out of 3 independent experiments, tested in duplicate. Two-way ANOVA, Bonferroni post hoc test. (**H**) Fluorescence of RAW 264.7 cells exposed to anakinra and stained with DCF-DA or MitoSOX red. (**I**) RAW 264.7 cells were starved or exposed to anakinra before visualization (magnified in the last column) of H_2_O_2_/mitochondria colocalization by staining with DCF-DA and MitoTracker red probes, respectively. Starvation was carried out in Earle’s balanced salt solution. DAPI was used to detect nuclei. Images were acquired using the Olympus BX51 fluorescence microscope. For *P* value, see Supplemental Figure 5.

To understand the molecular mechanisms behind our observation of IL-1R1–independent activity of anakinra, we assessed gene expression in purified C57BL/6 and *Il1r1^–/–^* alveolar macrophages exposed to anakinra in vitro to identify genes that are specifically influenced by anakinra in the absence of IL-1R1. RNA-Seq data analysis revealed that 1109 genes (Figure 2, A and B and Supplemental Table 1) were modulated by anakinra in both types of murine cells. Gene ontology (GO) functional classification analysis of differentially expressed genes revealed that 3 main biological processes were involved, i.e., biological regulation, response to stimulus, and response to stress (Supplemental Figure 4). Accordingly, genes involved in the oxidative stress and antioxidative defense pathways, known to be involved in the activation of autophagy (20), were significantly upregulated in *Il1r1^–/–^* cells by anakinra, mostly *Nox4,* followed by *Sod3* and *Gpx7*, among others (Figure 2C and Supplemental Table 2). In vivo, *Nox4*, more than *Sod3* or *Gpx7*, gene expression was significantly induced by anakinra in the lungs of infected *Il1r1^–/–^*mice along with the catalytic subunit *p22^phox^* gene (Figure 2D). NOX4 plays a pivotal role in autophagy (21). Unlike the other NOX isoforms, NOX4 directly produces H_2_O_2_ because of its unique E loop and intrinsic capacity to induce H_2_O_2_ and can be localized in mitochondria (22, 23). As a next step, we measured ROS production by anakinra in RAW 264.7 cells. In line with anakinra inducing NOX4 activity, anakinra did not induce mitochondrial (Figure 2E) or NADPH oxidase-dependent superoxide O_2_^–^ (Figure 2F) but induced mitochondrial H_2_O_2_, as revealed by (a) dihydrorhodamine 123 (DHR) fluorescence in the presence of allopurinol (that scavenges xanthine-oxidase–dependent ROS) but not MitoTEMPO (that scavenges mitochondrial ROS) (Figure 2G); (b) the positive immunostaining of anakinra-treated cells with 2’,7’-dichlorofluorescin diacetate (DCF-DA), a probe that reacts mainly with H_2_O_2_ to form a fluorescent compound but not MitoSOX (that detects mitochondrial O_2_^–^) (Figure 2H); and (c) the positive double immunostaining with MitoTracker red, a marker for functional mitochondria, and DCF-DA. Similar to what was observed in starvation conditions leading to H_2_O_2_ production that induces autophagy, all the DCF punctae colocalized with MitoTracker-stained structures (Figure 2I and Supplemental Figure 6 for colocalization indexes). These data strongly support mitochondria being the source of the H_2_O_2_ induced by anakinra. Experiments in vitro in purified *Il1r1^–/–^* alveolar macrophages and in vivo in infected *Il1r1^–/–^* mice confirmed that H_2_O_2_ production by anakinra was IL-1R1 independent and also occurred in the presence of IL-1R1 (Supplemental Figure 7).

### Anakinra promotes autophagy via mitochondrial H_2_O_2_.

**Figure 3:**
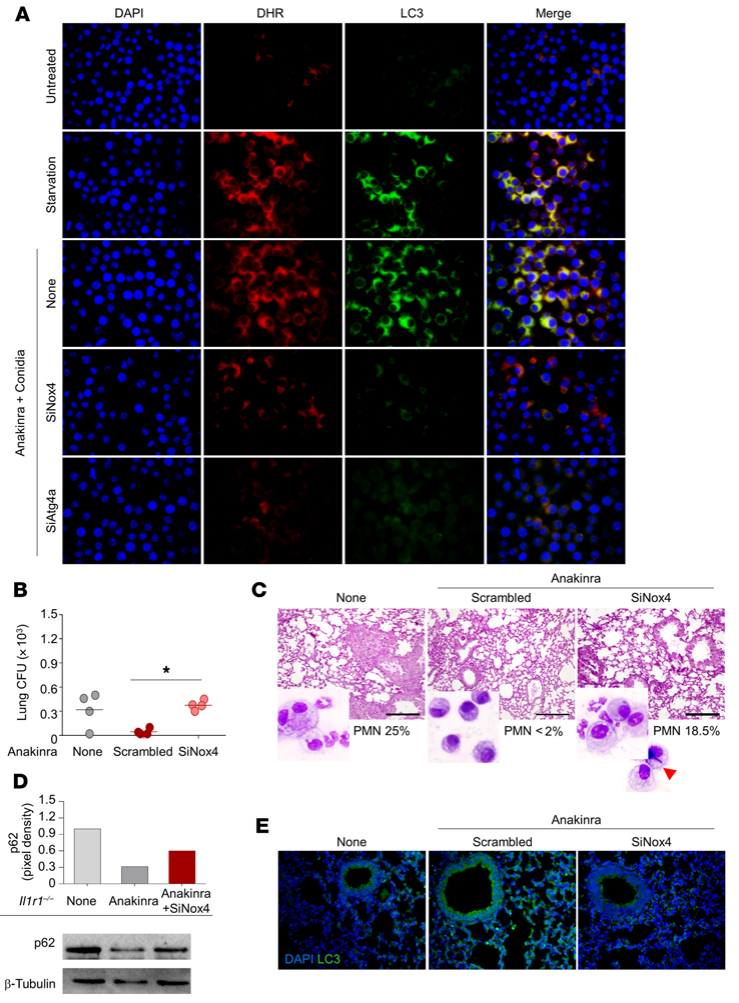
Anakinra promotes autophagy via mitochondrial H_2_O_2_. (**A**) Fluorescence images of EGFP-LC3–transfected RAW264.7 cells exposed to anakinra and *A*. *fumigatus* conidia for 4 hours alone or upon inhibition of *Atg4a* or *Nox4* by SiRNA. None, cells exposed to scrambled RNA. H_2_O_2_ was visualized using 10 μM DHR. Images were acquired using the Olympus BX51 fluorescence microscope and analySIS image processing software (Olympus). DAPI was used to detect nuclei. Data are from 1 representative out of 3 independent experiments. (**B**–**E**) *Il1r1^–/–^* mice were infected and treated with anakinra as in legend to Figure 1, administered SiNox4 or scrambled peptide, and assessed for fungal growth (log_10_ CFU) (**B**), histology [PAS staining] I, p62 immunoblotting (**D**), and LC3 immunofluorescence staining (**E**) in lung cell lysates or lungs. Insets in **C** show the bronchoalveolar lavage morphometry with the percentage of polymorphonuclear cells (PMNs) and an arrow indicating the appearance of fungi in SiNox4-treated mice. For CFU, lung staining, and immunoblotting, data are representative of 1 out of 2 independent experiments. Each independent in vivo experiment includes 4 mice per group. *P < 0.05, SiNox4-treated versus scrambled peptide. One-way ANOVA, Bonferroni post hoc test. Effectiveness of silencing was verified by quantitative RT-PCR analysis at 24 hours (Supplemental Figure 9).

A local rise in H_2_O_2_ in the vicinity of mitochondria leads to lipidation of Atg8, an essential step in the process of autophagy, via the redox-sensitive Atg4 cysteine protease (24). Atg4 enables the conversion of LC3-I to lipidated LC3-II, its insertion into the autophagosome, and the recycling of LC3-II after autophagosome–lysosome fusion. Given that anakinra did not affect the viability of the cells and actually retarded senescence (Supplemental Figure 8), which is induced by autophagy (25), we hypothesized that the H_2_O_2_ induced by anakinra may serve as a signaling molecule for autophagy. We evaluated H_2_O_2_/LC3 colocalization in starved cells and in anakinra-treated RAW 264.7 cells exposed to *Aspergillus* conidia while inhibiting Atg4a by RNA interference. Similar to starved cells, H_2_O_2_ colocalized with LC3-stained phagosome in anakinra-treated cells; blocking of *Nox4* or *Atg4a* prevented H_2_O_2_/LC3 colocalization induced by anakinra (Figure 3A and Supplemental Figure 9), suggesting that as in amino acid starvation (24), anakinra induces autophagy through the NOX4-dependent mitochondrial H_2_O_2_/Atg4 signaling pathway. Blocking *Nox4* also prevented anakinra-induced autophagy and control of fungal growth and inflammation in vivo in infected *Il1r1^–/–^* mice (Figure 3, B–E).

### Anakinra activates a xenobiotic sensing pathway via IDO1.

**Figure 4:**
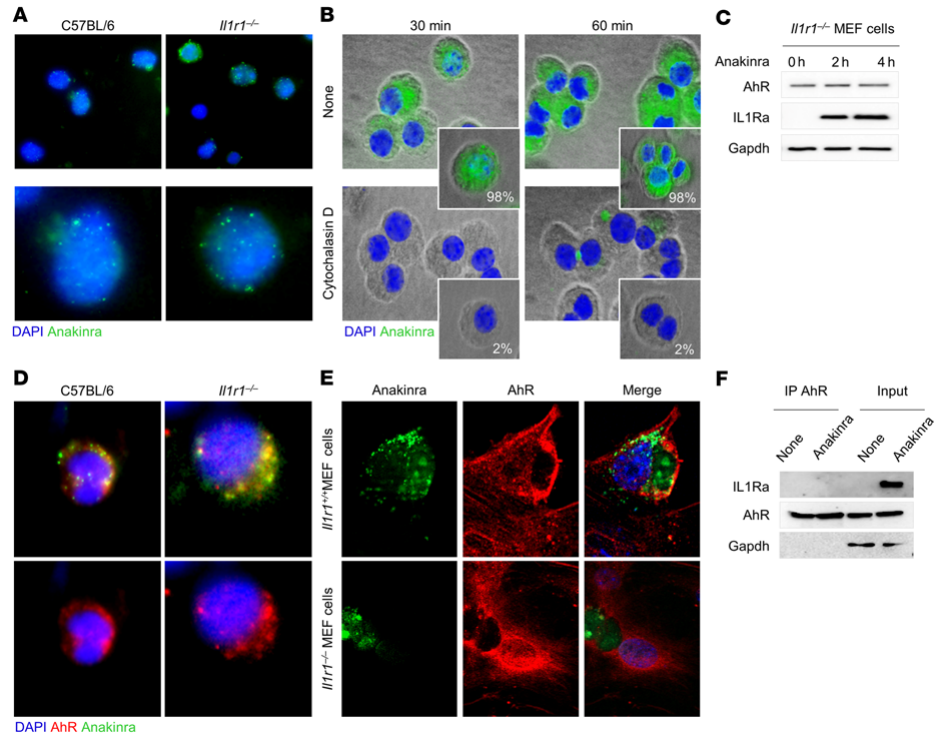
Anakinra colocalizes with AhR in *Il1r1^–/–^* cells. (**A**) Immunofluorescence imaging of cellular localization of FITC-anakinra in alveolar macrophages, isolated from naive C57BL/6 or *Il1r1^–/–^* mice, primed with 100 ng/mL LPS for 2 hours at 37°C, and exposed to 10 μg/mL FITC-anakinra for 60 minutes. (**B**) Immunofluorescence analysis of FITC-anakinra in *Il1r1^–/–^* cells exposed to FITC-anakinra at 30 and 60 minutes in the presence of 5 μM cytochalasin D. Insets, percentage positive cells. (**C**) *Il1r1^–/–^* MEF cells were treated with 10 μg/mL anakinra for 0, 2, and 4 hours and assessed for AhR and IL1Ra expression by immunoblotting with specific antibodies. (**D**) Immunofluorescence imaging of alveolar macrophages from naive C57BL/6 and *Il1r1^–/–^* mice primed with 100 ng/mL LPS for 2 hours at 37°C, exposed to 10 μg/mL FITC-anakinra for 60 minutes, and stained with anti-AhR antibody. (**E**) Immunofluorescence imaging of MEF cells stained with anti-AhR antibody and DAPI and exposed to 10 μg/mL FITC-anakinra for 60 minutes. (**F**) *Il1r1^–/–^* MEF cells were treated with 10 μg/mL anakinra, lysed, immunoprecipitated with anti-AhR antibody, and assessed for IL1Ra and AhR expression by immunoblotting with specific antibodies. None, untreated cells. Representative images, acquired with a fluorescent microscope (BX51), and immunoblots from 2 independent experiments are shown. DAPI was used to detect nuclei. Sections were examined using a Zeiss Axio Observer Z1.

**Figure 5:**
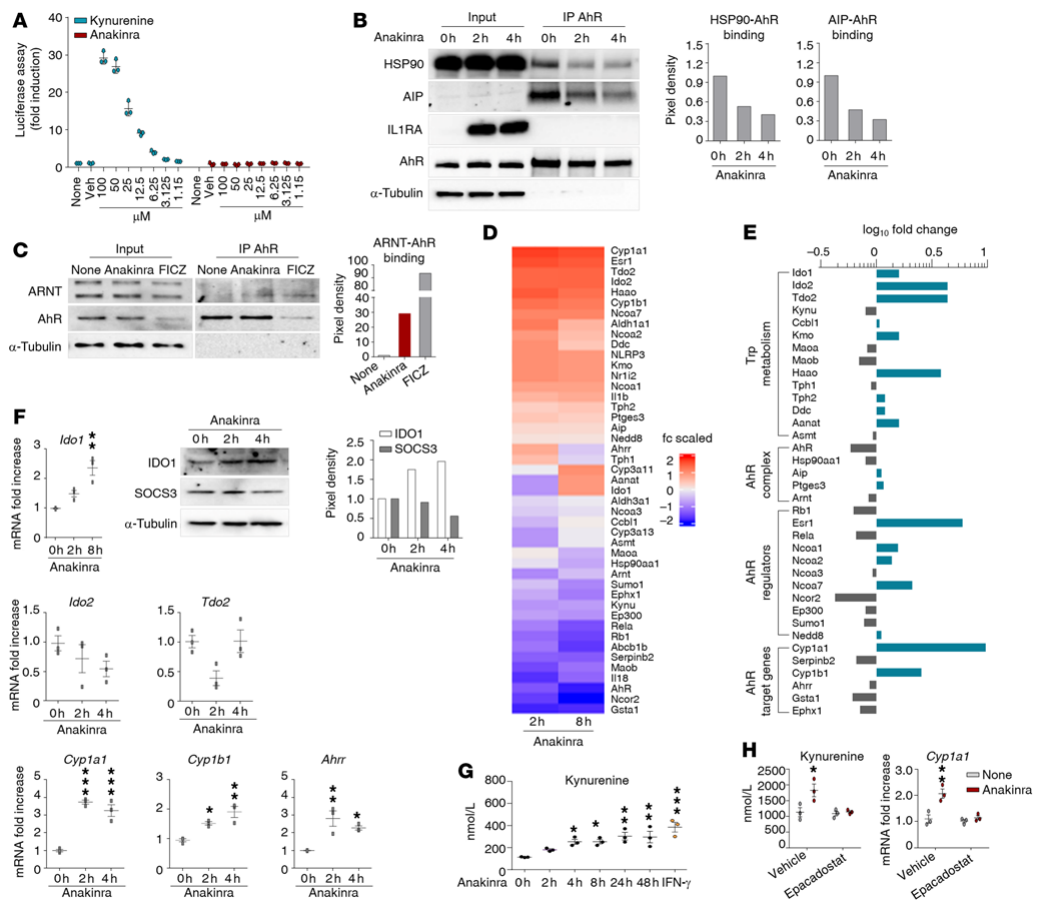
Anakinra activates a xenobiotic sensing pathway via IDO1. (**A**) H1L1 cells were treated with different doses of kynurenine and anakinra for 6 hours and assessed for luciferase assay. (**B**) Representative immunoblots of IL1RA, HSP90, AIP, and AhR and (**C**) ARNT and AhR in cell lysates in which AhR was immunoprecipitated from *Il1r1^–/–^* MEF cells treated with 10 μg/mL anakinra or 10 μM FICZ. In **B** and **C**, data are representative of 1 out of 2 independent experiments and the relative densitometric analysis are reported. (**D** and** E**) *Il1r1^–/–^* MEF cells were treated with 10 μg/mL anakinra for 2 and 8 hours and analyzed for gene expression by a custom QuantiGene plex gene expression assay. Fold changes are reported as heatmap for 2 and 8 hours (**D**) and histograms for 8 hours (**E**) (data are representative of 1 out of 2 independent experiments). (**F**) *Il1r1^–/–^* MEF cells were treated with 10 μg/mL anakinra and analyzed for mRNA expression of selected genes by RT-PCR (*n =* 3 independent samples) and protein expression of IDO1 and SOCS3 by immunoblotting (representative experiment). The relative densitometric analysis is reported. (**G**) *Il1r1^–/–^* MEF cells were treated with either 10 μg/mL anakinra for different times or 10 ng/mL IFN-γ as positive control for 48 hours and assessed for kynurenine production by ELISA. (**H**) *Il1r1^–/–^* MEF cells were treated with 10 μg/mL anakinra in the presence or absence of 10 μM epacadostat and assessed for kynurenine production by ELISA and *Cyp1a1* gene expression by RT-PCR (*n =* 3 independent samples). *P < 0.05, **P < 0.01, ***P < 0.001, treated versus untreated (None or 0 h) cells. One-way ANOVA, Bonferroni post hoc test.

Since anakinra induces autophagy via mitochondrial H_2_O_2_, independent of the IL-1R1, we explored potential mechanisms of cell entry not linked to IL-1R1. On assessing the uptake of FITC-anakinra in purified alveolar macrophages from C57BL/6 and *Il1r1^–/–^* mice by immunofluorescence imaging, we were able to detect anakinra intracellularly, particularly in *Il1r1^–/–^* cells (Figure 4A and confirmed by Western blot in Figure 4C), where the uptake was inhibited by cytochalasin D (Figure 4B). Therefore, consistent with previous findings (15), anakinra enters the cell independently of the IL-1R1 and might induce autophagy by interacting with intracellular mechanisms of autophagy. A general mechanism to which cells react to xenobiotics is via activation of cellular xenosensors that modulate gene expression that results in the generation of ROS and antioxidant proteins, thereby establishing a regulatory circuit and controlling processes such as apoptosis and autophagy (26). One such xenosensor is the aryl hydrocarbon receptor (AhR), which is known to modulate autophagy and inflammasome activity (27, 28). Preliminary experiments revealed that anakinra, in addition to *Nox4*, upregulated the expression of the AhR-target genes *Cyp1a1, Cyp1b1,* and *Ahrr* in *Il1r1^–/–^* total lung cells in vitro and in lungs of *Il1r1^–/–^* mice in vivo, albeit to a lesser extent compared with canonical AhR ligands (Supplemental Figure 10). Immunofluorescence staining revealed that anakinra colocalized with AhR in *Il1r1*^–/–^ cells, macrophages (Figure 4D and Supplemental Figure 6), and mouse embryonic fibroblast cells (Figure 4E and Supplemental Figure 6). Colocalization was higher in *Il1r1^–/–^* than C57BL/6 cells, likely because of the higher AhR protein expression in *Il1r1^–/–^* mice (Supplemental Figure 11). Despite the colocalization, the failure to show a direct association of anakinra with AhR by IP assay (Figure 4F) prompted us to assess how anakinra would engage AhR and activate its signaling. Consistent with the failure to detect a direct association with AhR, anakinra, as opposed to the AhR ligand kynurenine, did not activate AhR in a standard luciferase reporter assay (Figure 5A), thus confirming that it was not working as a direct AhR ligand. We then assessed whether anakinra may activate AhR via the production of putative intracellular AhR ligands. In this regard, we found that anakinra (a) promoted the ligand-dependent (29) dissociation of AhR from chaperons HSP90 and the AhR-interacting protein AIP (Figure 5B); (b) promoted the complex of AhR with the partner molecule ARNT (AhR nuclear translocator), required for promoter activity (Figure 5C); and (c) activated genes involved in tryptophan (trp) catabolism (*Ido1, Ido2, Tdo2, Haao,* and *Kmo*) together with the AhR-dependent genes (*Cyp1a1* and *Cyp1b1*) (Figure 5, D and E). Upon validation, beside the AhR-target genes, *Cyp1a1*, *Cyp1b1*, and the AhR repressor *Ahrr,* the IDO1 gene only was progressively upregulated by anakinra (Figure 5F). Of interest, early after anakinra exposure, SOCS3, which drives proteasomal degradation of IDO1 (30), was downregulated and IDO1 protein expression increased (Figure 5F), a finding suggesting that IDO1 activity may be promoted via transcriptional and posttranscriptional mechanisms. These findings indicate that anakinra may induce trp metabolism along the kynurenine pathway known to generate AhR ligands (31). We measured the levels of kynurenine and downstream metabolites in *Il1r1^–/–^*MEF cells exposed to anakinra and found that kynurenine (Figure 5G) and no other metabolites (Supplemental Table 3) were detected in a manner similar to the IDO1-inducer IFN-γ, a finding paralleling the activation of *Ido1* gene and downregulation of the *Kynu* gene with kynureninase activity (Figure 5E). Blocking IDO1 prevented both the production of kynurenine and the activation of AhR (Figure 5H), indicating that anakinra activates AhR via the IDO1-kynurenine pathway.

### Anakinra activates the AhR/Nox4/H_2_O_2_ cellular pathway in vivo.

**Figure 6:**
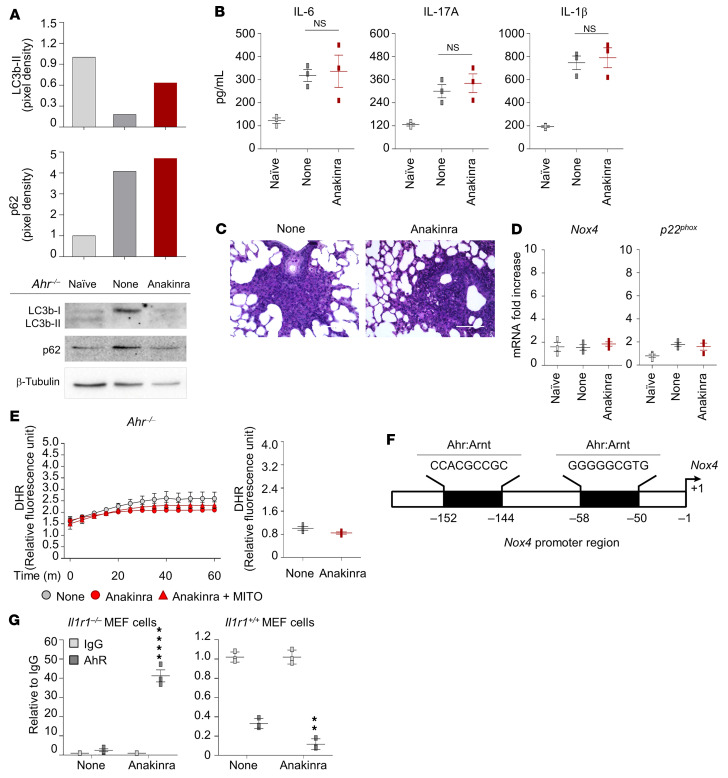
Anakinra promotes AhR transcriptional activity. (**A**–**C**) *AhR^–/–^* mice were infected and treated with anakinra as in legend to Figure 1 and assessed for immunoblotting of LC3b and p62 (**A**), cytokine production (ELISA) in lung homogenates (**B**), and lung histology (PAS staining) at 7 days after infection (**C**). Scale bar: 200 μm. Data represent 3 independent experiments. Each in vivo experiment includes 6 to 8 mice per group, pooled before analysis. NS, not statistically significant, treated versus untreated (None) cells. One-way ANOVA, Bonferroni post hoc test. (**D** and **E**) Ex vivo purified alveolar macrophages and total lung cells from *Ahr^–/–^* mice infected and treated with anakinra were assessed for *Nox4* and *p22^phox^* expression (RT-PCR) (*n =* 3 independent samples) (**D**) and H_2_O_2_ production (DHR staining) (*n =* 2 independent samples) (**E**). (**F**) Illustration of predicted binding sites from the ALGGEN-PROMO database and the Eukaryotic Promoter Database. (**G**) *Il1r1^–/–^* and *Il1r1^+/+^* MEF cells were treated with 10 μg/mL anakinra. ChIP assay was performed with AhR antibody. IgG was used as negative control. qPCR was conducted at the promoter regions of *Nox4*. Data are technical replicates of 1 representative out of 2 independent experiments.

The role of AhR was confirmed by further studies showing that anakinra failed to promote autophagy or decrease IL-1β, IL-6, and IL-17A production and tissue inflammation in *Ahr*^–/–^ mice (Figure 6, A–C). Moreover, neither *Nox4*/*p22^phox^* (Figure 6D) nor mitochondrial H_2_O_2_ production (Figure 6E) were induced by anakinra in these mice, further proving that induction of NOX4 and mitochondrial H_2_O_2_ by anakinra is dependent on AhR. By using ChIP-PCR assay, we were able to demonstrate that AhR binds to the binding site predicted by the ALGGEN-PROMO database and the Eukaryotic Promoter Database of the *Nox4* promoter in *Il1r1^–/–^* cells (Figure 6, F and G). Thus, anakinra enters myeloid cells through an actin-dependent fluid pinocytosis and promotes AhR-dependent Nox4 transcription independent of IL-1R1, which in turn induces autophagy via Atg4. An AhR/Nox4/H_2_O_2_ cellular pathway interfering with AhR endogenous regulation has already been shown (32).

### Anakinra restores functional cellular proteostasis via autophagy in CF.

**Figure 7:**
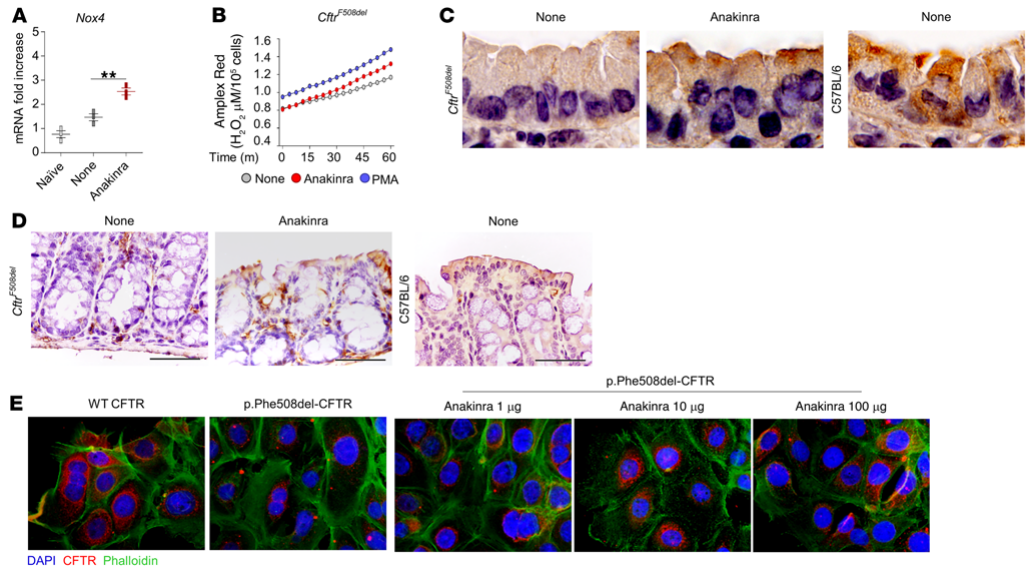
Anakinra restores cellular proteostasis in CF. (**A**–**D**) *Cftr^F508del^* mice were infected (i.n.) with live *A*. *fumigatus* conidia and treated with anakinra 10 mg/kg (i.p.) for 6 days. (**A**) *Nox4* gene expression in total lung cells at 7 dpi (*n =* 3 independent samples). None: infected, untreated mice. **P < 0.01, anakinra-treated versus untreated (None) mice. One-way ANOVA, Bonferroni post hoc test. (**B**) Amplex red fluorescence in purified alveolar macrophages from *Cftr^F508del^* mice after stimulation with 10 μg/mL anakinra or 10 ng/mL PMA for 4 hours at 37°C. For *P* values, see Supplemental Figure 5. Two-way ANOVA, Bonferroni post hoc test. (**C**) Lung and (**D**) small intestine expression of CFTR in mice infected and treated as above by IHC staining with anti-CFTR CF3 antibody (scale bar: 200 μm). Sections are representative of 3 independent experiments with *n =* 6 mice per group. C57BL/6 mice are shown as control. Images were acquired high-resolution microscopy (Olympus DP71 using ×100 objective). (**E**) Immunofluorescence staining of CFTR in CFBE41o^–^ cells stably transfected with WT or mutant CFTR and treated with different doses of anakinra for 4 hours at 37°C. Staining was done with the anti-CFTR CF3 antibody followed by Alexa Fluor 555 and Alexa Fluor 488 anti-phalloidin for F-actin labeling. Data are representative of 1 out of 2 (**E**) or 3 (**A**–**D**) independent experiments.

**Figure 8:**
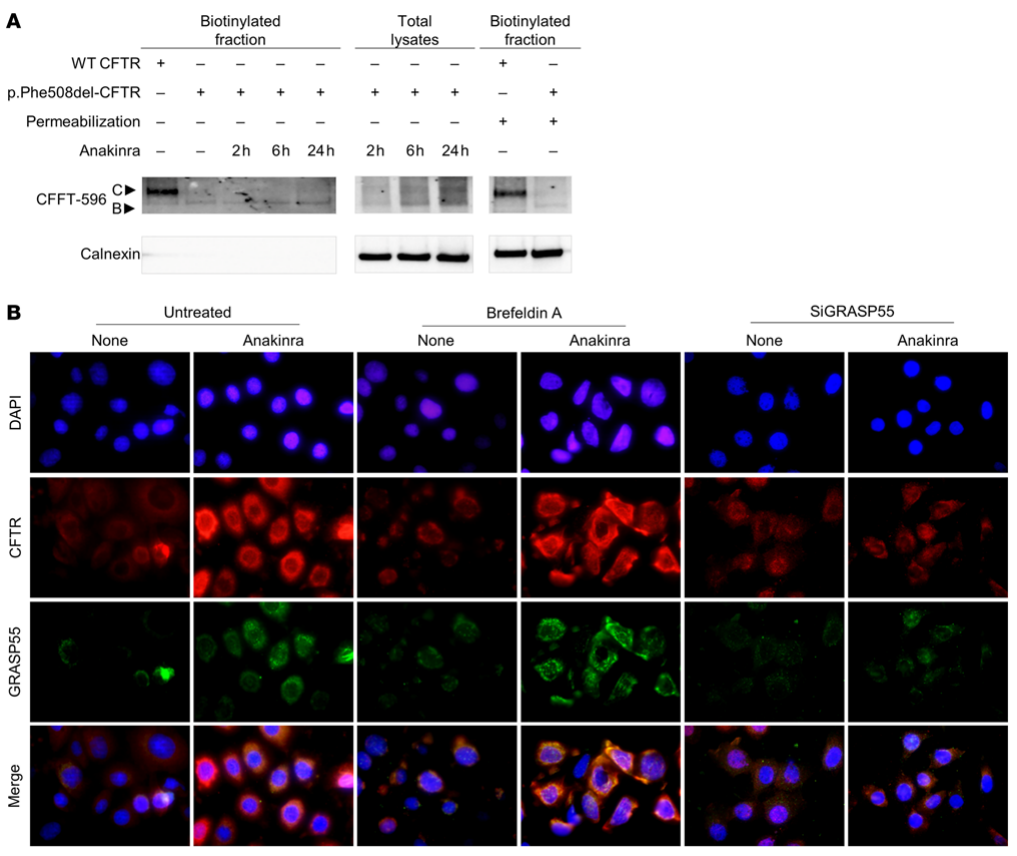
Anakinra increases surface expression of p.Phe508del-CFTR via the unconventional secretory pathway. Cell surface expression of CFTR in HEK293 cells transiently transfected with HA-p.Phe508del-CFTR and HA-WT-CFTR pCDNA3.1 plasmids and treated for 2, 6, or 24 hours with 10 μg/mL of anakinra at 37°C (**A**). Cells were immunoblotted with the anti-CFTR 596 and anti-calnexin antibodies after cell surface biotinylation and incubation with avidin solution. (**B**) Immunofluorescence staining of CFTR and GRASP55 in p.Phe508del-CFTR–transfected CFBE41o^–^ cells treated with 10 μg/mL anakinra or vehicle (None) at 37°C. Cells were pretreated with GRASP55 SiRNA or brefeldin A for 24 or 6 hours, respectively, at 37°C. Nuclei were counterstained with DAPI. Data are representative of 3 independent experiments.

**Figure 9:**
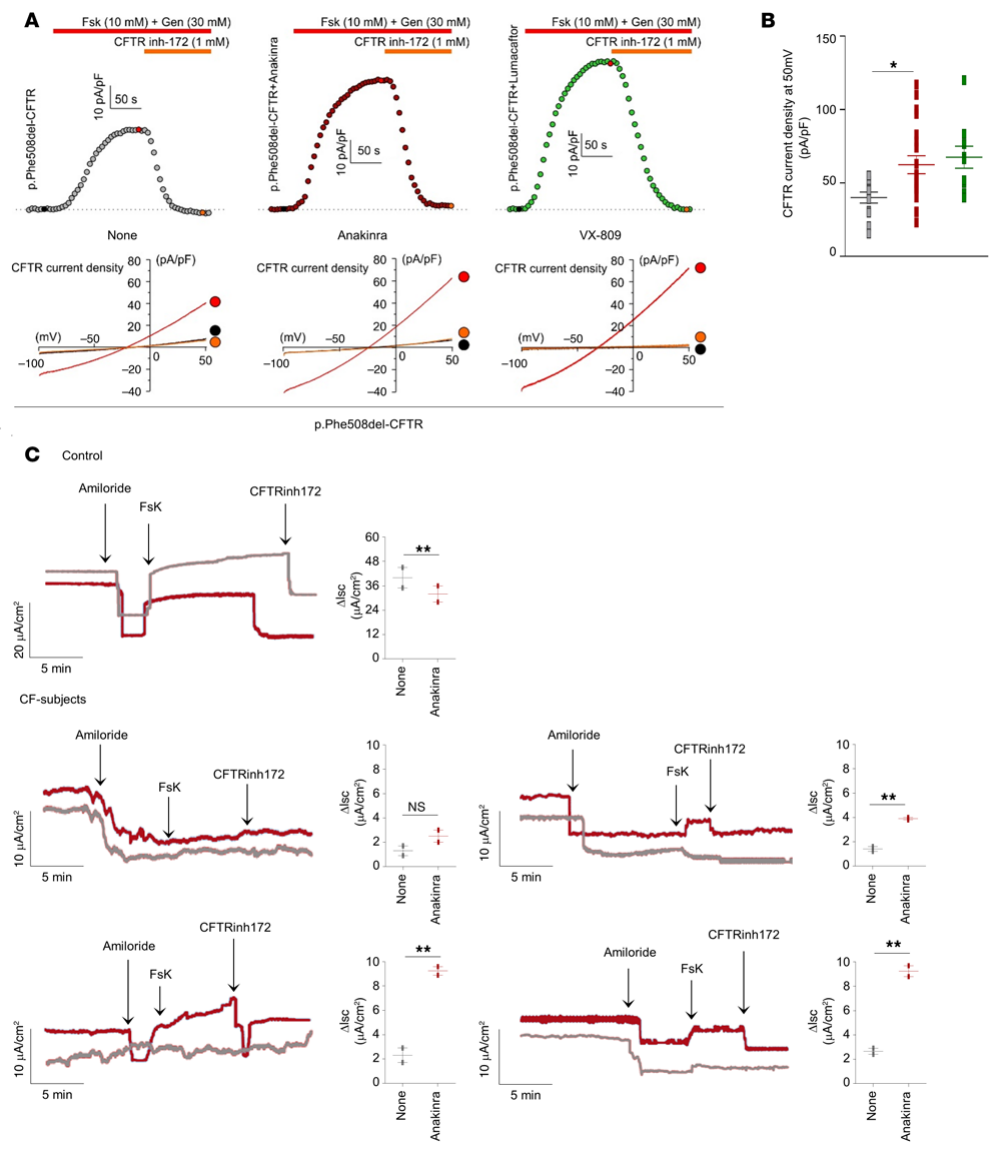
Anakinra increases CFTR activity. (**A**) Time course of whole-cell CFTR current densities induced by 10 μM forskolin (Fsk) + 30 μM genistein (Gen) at +50 mV in p.Phe508del-CFTR–transfected FRT cells treated with 10 μg/mL anakinra, 3 μM VX-809, or DMSO (None) for 4 hours at 37°C followed by blockade with CFTR inhibitor 172 (CFTR inh-172). The horizontal bars indicate the time period of drug application. Inset, current ramps from −100 mV to +50 mV (holding potential, −40 mV) in control condition (black trace), after application of Fsk + Gen (red trace), and after application of 1 μM CFTRinh-172 (orange trace). (**B**) Mean ± SEM CFTR current density measured at +50 mV induced by Fsk and Gen in cells treated with anakinra (*n =* 20), VX-809 (*n =* 10), or vehicle (*n =* 12). *P < 0.05, anakinra versus untreated (None), 1-way ANOVA, Bonferroni post hoc test. (**C**) CFTR-dependent chloride secretion measured by means of Fsk-induced increase in the Isc in HBE cells from 4 patients with CF and 1 representative control treated with anakinra for 4 hours at 37°C and mounted in Ussing chambers in the presence of CFTR inhibition (CFTR inh-172) and amiloride. **P < 0.01, anakinra-treated versus untreated (None) cells. NS, not statistically significant. Student’s *t* test. Data are from 2 experiments.

To demonstrate the clinical relevance of this newly identified mechanism, we used preclinical models of CF, a prototypical disease of dysregulated proteostasis that is linked to defective autophagy and aberrant inflammasome-mediated inflammation (14). We investigated whether anakinra, in addition to its receptor-dependent activity in CF (14), would affect trafficking of CFTR via secretory autophagy, a form of nondegradative autophagy that participates in the unconventional secretion of cytoplasmic entities (33) and involves an oxidative stress (34). We used 2 complementary preclinical models involving homozygous *F508del-Cftr* C57BL/6 mice (referred to as *Cftr^F508del^* mice) treated with anakinra in vivo and human cells bearing the p.Phe508del-CFTR mutation exposed to anakinra in vitro. We found that anakinra promoted *Nox4* gene expression (Figure 7A) and H_2_O_2_ production (Figure 7B) and restored CFTR expression in the lung (Figure 7C) and small intestine (Figure 7D) of homozygous *Cftr^F508del^* mice. Moreover, anakinra increased membrane expression of p.Phe508del-CFTR on p.Phe508del-CFTR–transfected CFBE41o^–^ cells as revealed by immunofluorescence staining (Figure 7E). Immunoblots (Figure 8A) revealed that at 37°C, anakinra induced the core glycosylated band B to a greater extent than the mature band C of CFTR, an effect suggesting the occurrence of the unconventional secretory pathway that, bypassing the Golgi, takes immature CFTR directly at the plasma membrane (35). Indeed, anakinra promoted secretory autophagy involving GRASP55, a protein mediating unconventional secretion of CFTR in the condition of ER stress blockade (35). Figure 8B shows that colocalization of CFTR with GRASP55 was greatly promoted by anakinra and brefeldin A and greatly reduced upon GRASP55 inhibition by SiRNA. The rescuing activity of anakinra correlated with an increased chloride ion channel function of the rescued p.Phe508del-CFTR to an extent similar to that obtained with the corrector VX-809 (Figure 9, A and B). In a more relevant setting, anakinra increased the chloride channel activity in HBE cells from patients homozygous for the p.Phe508del-CFTR mutation. Ussing chamber tracings revealed that anakinra promoted, although to a variable degree, the forskolin-induced increase of the chloride current (Isc) in 3 out of 4 patients and not in the control group (Figure 9C), an effect that was sensitive to CFTR inhibition.

## Discussion

The proteostasis network has established a new paradigm for deconvolution of disease mechanism and therapeutic management (2, 4). However, since targeting all the myriad defects individually could be quite challenging, identifying a druggable pathway that controls disease-promoting processes may overcome the targeted delivery that is a critical factor influencing efficacy. This study showed that anakinra paired with xenobiotic-induced redox signaling to promote autophagy and proteostasis. As such, the study represents a proof-of concept demonstration of how the knowledge of proteostatic pathways directing lung function may improve opportunities for strategies to reduce lung injury and disease, as already pointed out (6). While not acting as a direct AhR ligand, anakinra promoted the generation of intracellular AhR ligands, such as kynurenine, via IDO1, whose activity was promoted via transcriptional and posttranscriptional mechanisms. Both mechanisms are supported by the ability of anakinra to inhibit IL-6 and SOCS3, both driving proteasomal degradation of IDO1 (30), in mice (this study) and in humans (36) and by the ability of AhR to activate IDO1 transcription (37). Thus, the regulation of IDO1 degradation could be the initial working mechanism of anakinra that subsequently exploits the positive IDO1/kynurenine/AhR feedback loop that may limit inflammation in the condition of IL-1R1 deficiency. The finding that both kynurenine level (Supplemental Table 3) and AhR expression (Supplemental Figure 11) were increased in the condition of IL-1R1 deficiency supports this conclusion.

AhR transcriptionally activates NOX4, a critical mediator of autophagy via H_2_O_2_ (34). Besides the ER, NOX4 also localizes to the inner mitochondria membrane, where it functions as energetic sensor in this organelle (38). The mitochondrial localization of NOX4 is of interest because anakinra is known to induce mitochondrial SOD2 among the H_2_O_2_ scavengers (39). Given that SOD2 protects against mitochondrial oxidative damage and mitochondrial dysfunction (40), this implies that anakinra, by acting as a mitochondria-targeted antioxidant agent, can protect against oxidant- and mitochondrial-related disorders. Thus, the promotion of autophagy through a redox-signaling pathway involving mitochondrial NOX4 activity is a potentially novel mechanism through which lung proteostasis is achieved and its promotion by anakinra may broaden the clinical implications.

Here, we found that by coupling mitochondrial redox balance to autophagy, anakinra improved the proteostasis network in the CF lung and promoted surface expression of functional CFTR through conventional and unconventional secretion pathways. We have already shown that disease susceptibility in murine CF and microbial colonization in patients with CF occurred in conditions of genetic deficiency of IL-1Ra, was associated with pathogenic inflammasome activity, and could be rescued by administration of anakinra (14, 41). Thus, within the emerging therapeutic option of targeting the NLRP3 inflammasome as antiinflammatory strategy in CF, the finding that anakinra could have additional beneficial effects beyond NLRP3 inhibition (17) and IL-1 blockade (42) further reinforces its repurposing as a therapeutic agent in CF. This is emphasized by a recent trial that will investigate the effects of anakinra in CF (ClinicalTrials.gov NCT03925194).

By showing the involvement of the xenobiotic sensing AhR pathway, this study provides further foundations for the therapeutic potential of anakinra. Although the extent to which the IL-1R1–dependent and independent pathways are intertwined in response to anakinra in vivo is likely dependent on many factors, including the levels of expression of IL1R1 and AhR, and the specific cell type, the different intracellular pathways activated by anakinra may account for its multifaceted activity and off-target effects and predict the contingency of anakinra’s effects on environment and epigenetic factors affecting AhR activity. In line with our findings, a recent study suggested that IL-1Ra pairs with xenobiotic-induced immune signaling in human disease (43). Thus, at the intersection of mitochondrial oxidative stress, autophagy, and proteostasis, anakinra could be exploited in a variety of chronic human diseases associated with dysregulated redox status, defective proteostasis, and autophagy, such as aging and COVID-19 (44, 45).

In conclusion, this study provides further foundations for the possible use of anakinra in lung inflammatory diseases and suggests that the repurposing of anakinra at the intersection of mitochondrial oxidative stress, autophagy, and proteostasis in human diseases warrants further investigation.

## Methods

### Mice, infections, and treatments.

Male and female, 8- to 10-week-old, C57BL6 WT mice were purchased from the Jackson Laboratory. Breeding pairs of homozygous *Il1r1^–/–^* (Charles River Laboratories) and *Ahr^–/–^* mice raised on C57BL6 background were bred under specific pathogen–free conditions at the breeding facilities of the University of Perugia, Perugia, Italy. B6.129-Ahrtm1Bra/J Ahr-deficient (*Ahr*^–*/*–^) mice were supplied by B. Stockinger (MRC National Institute for Medical Research, London, United Kingdom). CF mice homozygous for the Phe508del-*Cftr* allele, which had been backcrossed for 12 generations to the C57BL/6 strain (Cftr^tm1EUR^ Phe508del, abbreviated *Cftr^F508del^*), were obtained from B. Scholte (Erasmus Medical Center, Rotterdam, Netherlands; ref. 46). These mice were provided with a special food consisting of an equal mixture of SRM-A (Arie Blok, Woerden) and Teklad 2019 (Harlan Laboratories) and water acidified to pH 2.0 with HCl and containing 60 g/L PEG 3350, 1.46 g/L NaCl, 0.745 g/L KCl, 1.68 g/L NaHCO_3_, and 5.68 g/L Na_2_SO_4_. Newborn mice were genotyped by cutting a small piece of tail 12 days after birth. Mice were anesthetized in a plastic cage by inhalation of 3% isoflurane (Forane, Abbott) in oxygen before i.n. instilling 2 × 10^7^ *A*. *fumigatus* (*Af293*) resting conidia per 20 μL of saline. For *P*. *aeruginosa* infection, clinical *P*. *aeruginosa* strain, isolated from a patient, was obtained from the Diagnostic Unit of Microbiology from the University of Perugia. The bacteria were grown for 3 hours to reach the exponential phase. Next, the bacteria were pelleted by centrifugation (2700*g*, 15 minutes) and washed twice with sterile PBS, and the OD of the bacterial suspension was adjusted by spectrophotometry at 600 nm. The intended number of CFUs was extrapolated from a standard growth curve. Appropriate dilutions with sterile PBS were made to prepare the inoculum before i.n. instilling 3 × 10^7^ CFU per mice. Quantification of fungal growth was done as described (17). Bronchoalveolar lavage (BAL) fluid was collected in a plastic tube on ice and centrifuged at 400*g* at 4°C for 5 minutes. For differential BAL fluid cell counts, cytospin preparations were made and stained with May-Grünwald Giemsa reagents (Sigma-Aldrich). For histology, paraffin-embedded sections were stained with periodic acid–Schiff (PAS). Mice were treated (i.p.) with 10 mg/kg anakinra reconstituted in sterile water daily for 6 consecutive days beginning the day of the infection. Mice were euthanized a day after treatment. Infections were performed under isoflurane anesthesia, and all efforts were made to minimize suffering.

### Cells and treatments.

RAW 264.7 cells were from ATCC (TIB-71). MEF cells were generated as described (47). HEK293 cells were from ATCC (CRL-1573). HBE cells, homozygous for the p.Phe508del mutation, and isogenic WT cells were obtained from lung transplants (individuals with CF) or lung resections (control) (provided by LJ Galietta, Italian Cystic Fibrosis Foundation, Verona, Italy). Cells were maintained at 37°C in a humidified incubator in an atmosphere containing 5% CO_2_. Stable lentiviral-based transductions of the parental CFBE41o^–^ cells, homozygous for the p.Phe508del-CFTR mutation (48), with either WT CFTR or p.Phe508del-CFTR, were provided by LJ Galietta. The transduced CFBE41o^–^ cells were maintained in MEM supplemented with 50 U/mL penicillin, 50 μg/mL streptomycin, 2 mM l-glutamine, 10% FBS, and 1 μg/mL blasticidin (WT CFTR) or 2 μg/mL puromycin (p.Phe508del-CFTR) in a 5% CO_2_ and 95% air incubator at 37°C. To establish polarized monolayers, CFBE41o^–^ cells were seeded on 24 mm-diameter Transwell permeable supports (0.4 mm pore size; Corning Corp) at 2 × 10^6^ cells/well and grown in an air-liquid interface culture at 37 °C for 6–9 days and then at 27°C for 36 hours. Fisher rat thyroid (FRT) epithelial cells, provided by LJ Galietta, were cultured on 60 mm Petri dishes with Coon’s modified F12 containing 5% serum, 2 mM l-glutamine, 50 U mL^–1^ penicillin, and 50 μg mL^–1^ streptomycin. Cells stably transfected with CFTR plasmid were provided by LJ Galietta. Total lung cells and alveolar macrophages were obtained from C57BL6 and *Il1r1^–/–^* mice as described (14).

Anakinra (Kineret, Amgen Europe) was diluted in PBS 1× (vehicle). Cells were incubated with different doses of anakinra for up to 24 hours. FITC-anakinra was obtained with the FITC Conjugation Kit (Abcam), as per the manufacturer’s instructions. Truncated anakinra was obtained as described below. In selected experiments, cells were treated with the AhR agonists 5,11-dihydro-indolo[3,2-b]carbazole-6-carboxaldehyde, 6-formylindolo[3,2-b]carbazole (FICZ, Sigma-Aldrich), 2-(1’H-indole-3′-carbonyl)-thiazole-4-carboxylic acid (ITE, Sigma-Aldrich), and kynurenine (Sigma-Aldrich), or the IDO1 inhibitor epacadostat (SelleckChem).

### Measurements of AhR activation.

To assess the activation of AhR, we used mouse hepatoma cells (H1L1.1c2), containing the stably integrated AhR xenobiotic responsive element driven by a firefly luciferase reporter plasmid, pGudLuc6.1 (49). Cells were seeded in 96-well plates at a density of 0.1 × 10^6^ cells in 200 μL. After 12 hours at 37°C, cells were stimulated for 6 hours with increasing concentrations of kynurenine or anakinra before lysis. Luciferase assays were performed using luciferase reporter assay kit (Promega). The *Renilla* luciferase activity was measured, and results are presented as fold induction.

### Limited proteolysis.

The proteolytic cleavage of anakinra was performed as described (50). Briefly, a stock protein solution at 10 mg/mL concentration was diluted in PBS pH 7.0 at 1 mg/mL and incubated at 25°C with proteinase K (P5568, Sigma-Aldrich) at an enzyme/substrate ratio of 1:100 for 1 hour. The reaction was stopped by addition of PMSF at 2 mM concentration. As a control, in a parallel sample, PMSF was added from the beginning. Protein cleavage was evaluated by SDS-PAGE analysis (Supplemental Figure 2A). The overall folding of cleaved anakinra was evaluated by registering intrinsic fluorescence emission spectra before and after proteinase K treatment using a Jasco J-715 spectropolarimeter equipped with a thermostatically controlled cell holder. Protein emission spectra were taken from 300 to 500 nm (excitation at 280 nm) with both the excitation and the emission slits set to 5 nm. All spectra were corrected by subtracting the emission spectrum of the blanks, i.e., of samples containing all reagents except anakinra (Supplemental Figure 2B).

### Genome sequencing, assembly, and analysis.

Total RNA was extracted from C57BL/6 and *Il1r1^–/–^* alveolar macrophages purified and stimulated in vitro with 10 μg/mL of anakinra for 4 hours at 37°C. The total RNA samples were first treated with DNase I to degrade any possible DNA contamination. Then, the mRNA was enriched by using oligo(dT) magnetic beads. Mixed with the fragmentation buffer, the mRNA was fragmented into short fragments (about 200 bp). Then, the first strand of cDNA was synthesized by using random hexamer-primers. Buffer, dNTPs, RNase H, and DNA polymerase I were added to synthesize the second strand. The double-strand cDNA was purified with magnetic beads. End reparation and 3′-end single nucleotide A (adenine) addition was then performed. Finally, sequencing adaptors were ligated to the fragments. The fragments were enriched by PCR amplification. During the quality control step, Agilent 2100 Bioanalyzer and ABI StepOnePlus Real-Time PCR System were used to qualify and quantify of the sample library. The library products were ready for sequencing via Illumina HiSeq 2000 or another sequencer when necessary. Illumina sequencing using the HiSeq 2000 platform was performed at the Beijing Genomics Institute, Shenzhen, China (www.genomics.cn/index.php) according to the manufacturer’s instructions. Functional annotation by GO (http://www.geneontology.org) was analyzed by Blast2go (51) and WEGO (52) software. The gene expression data set has been deposited in NCBI’s Gene Expression Omnibus database (accession number GSE188809).

### Autophagy.

RAW264.7 cells were treated with 50 μM rapamycin (LC laboratories), *A*. *fumigatus* swollen conidia (1:1 ratio), and/or 10 μg/mL anakinra, full-length and or truncated, at 37°C for 4 hours. In selected experiments, 100 nM bafilomycin A1 (Sigma-Aldrich) or 100 μM chloroquine (Sigma-Aldrich) was added. After treatment, cells were fixed in 2% formaldehyde for 15 minutes at room temperature and permeabilized in blocking buffer containing 3% BSA and 0.5% Triton X-100 in PBS. The cells were then incubated at 4°C with the primary antibody anti-LC3b (Abcam, ab48394). After extensive washing with PBS, the slides were incubated at room temperature for 60 minutes with goat anti-rabbit antibody to LC3 followed by Alexa Fluor 488 (Molecular Probes). DAPI (Molecular Probes) was used to counterstain nuclei. Optical sections were examined using a Zeiss Axio Observer Z1 inverted microscope equipped with ApoTome filter and Axiocam MRm camera detection system. The number of LC3 puncta was quantified with ImageJ (NIH) (53) and expressed as number of puncta/cell. In selected experiments, RAW264.7 cells were seeded in 100 mm Petri dish (3.5 × 10^6^) and transfected with the EGFP-LC3 plasmid (Addgene) using ExGen 500 in vitro transfection reagent (Fermentas) for 48 hours following the manufacturer’s instructions. Transiently transfected RAW264.7 cells were exposed to *A*. *fumigatus* swollen conidia (1:1 ratio) and 10 μg/mL anakinra and incubated for 4 hours at 37° C in 5% CO_2_ as described (17). Starvation was carried out in Earle’s balanced salt solution. Cultures growing on coverslips were observed at ×100 magnification with the Olympus BX51 fluorescence microscope using an FITC filter. For autophagy on lung cells, purified 1 × 10^6^ alveolar macrophages from naive mice were stimulated on glass slides in 24 multi-well plates with anakinra and/or *Aspergillus* conidia as above for 4 hours at 37°C in 5% CO_2_. Cells were incubated with 1:200 diluted anti-LC3 antibody (Cell Signaling Technology, 2775S) overnight at 4°C in PBS containing 3% normal BSA, incubated with anti-rabbit PE secondary antibody (Sigma-Aldrich), and fixed for 20 minutes in PBS containing 4% paraformaldehyde. Images were acquired using the Olympus BX51 fluorescence microscope with a ×100 objective and analySIS image processing software (Olympus). DAPI was used to detect nuclei.

### SiRNA design and delivery.

Predesigned SiRNA against *Atg4a* (MMC.RNAI.N174875.12.1), *Nox4* (mm.Ri.Nox4.13.1), and *GORASP2* (coding for GRASP55) (duplex name: hs.Ri.GORASP2.13.1) were purchased from Integrated DNA Technologies (IDT) (TEMA Ricerca). Cells were incubated for 24 hours (as indicated by preliminary experiments performed at 12, 24, or 48 hours) at 37°C in 5% CO_2_ with specific SiRNA using Lipofectamine LTX reagent (Invitrogen) following the manufacturer’s instructions. Effectiveness of silencing of specific targets was verified by real-time PCR (RT-PCR) analysis at 24 hours (Supplemental Figure 9). For in vivo experiments, each mouse received i.n. administration of 10 μg/kg unmodified SiRNA or equivalent dose of nonspecific control SiRNA duplex in a volume of 20 μL of duplex buffer (IDT). I.n. SiRNA was given once the day before infection and 2 days after infection (54).

### Western blot analysis and IP assay.

For Western blot and IP assay, lung and cell lysates were harvested after treatment at the indicated time, washed twice with cold PBS (Sigma-Aldrich), and lysed in RIPA buffer (Tris/HCl at pH 8.0, 50 mM, NaCl 150 mM, SDS 0.1%, sodium deoxycholate 1%, Triton X-100 1%), supplemented with protease inhibitor cocktail (PIC, Roche) and PMSF (Sigma-Aldrich). For IP, lysates were incubated overnight at 4°C with the specific antibody followed by 2 hours of incubation with protein G- or protein A-Sepharose beads (GE Healthcare). Immune complexes were washed 4 times in washing buffer and boiled in 2× sample buffer. The proteins were separated by electrophoresis on SDS-PAGE and detected using specific antibodies against LC3b I and II (Cell Signaling Technology, 2775S), p62 (Cell Signaling Technology, 5414S), IDO1 (cv152, as described in ref. 31), AhR (Invitrogen, MA1-514), IL-1Ra (Invitrogen, PA5-21776), ARNT (Cell Signaling Technology, 5537), HSP90 (Proteintech, 13171-1-AP), AIP (Proteintech, 18176-1-AP), and SOCS3 (Cell Signaling Technology, 2923S). Normalization was performed with β-actin, α-tubulin, β-tubulin, or Gapdh antibodies (Sigma-Aldrich, A3853, T9026, T4026, G8795). Protein signal intensities were quantified by densitometric analysis using ImageJ software. See complete unedited blots in the supplemental material.

### ROS determination and fluorescence microscopy.

For ROS determination, RAW 264.7 cells or ex vivo purified lung macrophages were plated in HBSS buffer with Ca^2+^ and Mg^2+^ and without phenol red and exposed to 10 μg/mL anakinra or 10 ng/mL PMA (phorbol 12-myristate 13-acetate, Sigma-Aldrich) for 4 hours at 37°C. We used dihydroethidium (DHE, Molecular Probes) to detect intracellular and extracellular superoxide production by phagocytic NADPH oxidase (55); MitoSOX red (Molecular Probes) to detect mitochondrial superoxide; Amplex Red (Invitrogen) for H_2_O_2_; and DHR (Sigma-Aldrich) to detect intracellular ROS, mainly O_2_− and H_2_O_2_ (56). As inhibitors, we used diphenyleneiodonium (DPI, Sigma-Aldrich) to inhibit NADPH oxidase, MitoTEMPO (Enzo Life Science) to scavenge mitochondrial ROS, and allopurinol (Sigma-Aldrich) to scavenge xanthine oxidase–dependent O_2_− and H_2_O_2_. Next, 10 μM DHR, 50 μM DHE, and 5 μM MitoSOX red were added to cells exposed to PMA or anakinra for the last 30 minutes at 37°C, after which the reagents were washed out. Cells were preincubated with 50 μM MitoTEMPO for 60 minutes before the addition of anakinra. Then, 50 μM allopurinol or 2 μg/mL DPI was added for 20 or 60 minutes at 37°C. The DHR (ex 100/em 530), DHE (ex 518/em 605), and MitoSOX red (ex 510/em 580) signals were measured by a Tecan Infinite 200 microplate reader. During the measurement, the cells were maintained at 37°C. For fluorescence microscopy, cells were fixed in PBS containing 4% paraformaldehyde. For colocalization, H_2_O_2_ was visualized using 1 mM of DCF-DA (Molecular Probes), and mitochondria were stained with 200 nM of MitoTracker red (Molecular Probes). Because H_2_O_2_ is the major peroxide in cells, it is generally accepted that DCF is proportional to H_2_O_2_ concentration (57). Images were acquired using the Olympus BX51 fluorescence microscope with either a ×100 or ×40 objective and analySIS image processing software (Olympus). For anakinra entry and AhR cellular localization, cells were exposed to 10 μg/mL FITC-anakinra for 30 minutes, and optical sections were examined using a Zeiss Axio Observer Z1 inverted microscope equipped with ApoTome filter and Axiocam MRm camera detection system after staining with rabbit anti-AhR antibody (Santa Cruz Biotechnology, sc-101104) or DAPI to detect nuclei.

### ChIP assay.

MEF cells were grown on 100 mm plates incubated with 10 μg/mL anakinra for 4 hours. Cells were then recovered in PBS 1× with MgCl_2_ 1 mM, exposed to 1% formaldehyde at room temperature for 15 minutes to induce DNA crosslinking, washed, and then lysed with RIPA buffer containing 150 mM NaCl, 50 mM Tris pH 8.0, 1% Triton X-100, 0.5% sodium deoxycholate, 0.1% PIC, and PMSF. Cell lysates were sonicated to shear DNA into 200 to 500 bp fragments and subjected to IP using ChIP-grade anti-AhR monoclonal antibody (Thermo Fisher Scientific, MA1-513) at 4°C overnight. Normal mouse IgG (Sigma-Aldrich, 12-371) was used as the ChIP-negative control. The ChIP product was incubated with salmon sperm conditioned protein A Sepharose for 3 hours at 4°C to recover AhR-bound DNA. The resulting material was washed; collected in CHIP elution buffer containing 300 mM NaCl, 10 mM Tris pH 8.0, 0.5% SDS, and 5 mM EDTA pH 8.0; and then de-crosslinked, purified, and resuspended in ultrapure water. AhR binding to chromatin was quantified using qPCR with primers specific to the regulated gene promoter. The ChIP primers used for *Nox4* promoter detection were *Nox4:* ATGTCTGCAGCTGGACAGG and ACCGAAAGGAGCGATCAGT. All reactions were run in triplicate.

### Cell-surface biotinylation assay.

HEK293 cells were grown to 70% to 80% confluence in 6-well plates coated with poly-D-lysine (Sigma-Aldrich). Transient transfections with HA-p.Phe508del-CFTR and HA-WT-CFTR pCDNA3.1 plasmids were performed using Lipofectamine LTX Transfection Reagent (Invitrogen), according to the manufacturer’s instructions. The day after transfection, cells were treated for 2, 6, or 24 hours with 10 μg/mL of anakinra. Cells were then placed at 4°C and washed 3 times with PBS/CaCl_2_/MgCl_2_ (PBS + 2.5mM CaCl_2_, 1mM MgCl_2,_ pH 7.4). Plasma membrane proteins were then biotinylated by gently shaking the cells in a PBS/CaCl_2_/MgCl_2_ buffer containing EZ-Link Sulfo-NHS-SS-Biotin (Thermo Fisher Scientific) for 30 minutes. Some cells were permeabilized with 0.05% Triton X-100 in cold PBS/CaCl_2_/MgCl_2_ for 10 minutes before biotin treatment. After biotinylation, cells were washed extensively with quenching buffer (50 mM glycine in PBS/CaCl_2_/MgCl_2_) to remove excess biotin and then washed twice with PBS. The cells were then lysed and incubated overnight at 4°C with avidin solution (NeutrAvidin Plus UltraLink Resin, Pierce). Avidin-bound complexes were pelleted and washed 3 times by rotating the mixture for 5 minutes at 4°C. Biotinylated proteins were eluted in a 1× sample buffer, resolved by SDS-PAGE, electrotransferred, and immunoblotted with the anti-CFTR antibody 596 CFTR (provided by JR Riordan through a program of the Cystic Fibrosis Foundation Therapeutics [CFFT], Charlotte, North Carolina, USA) and anti-calnexin (Abcam, ab22595).

### Immunofluorescence and IHC.

LC3 or AhR immunofluorescence were done on lung sections by staining with anti-LC3b (Abcam, ab48394) or anti-AhR (Proteintech, 17840-1-AP) antibody and secondary labeled antibodies. For CFTR immunofluorescence on CFBE41o^–^ cells, cells were treated with 10 μg/mL anakinra at 37°C for 4 hours, fixed in 2% formaldehyde for 15 minutes at room temperature, and permeabilized in blocking buffer containing 5% FBS, 3% BSA, and 0.5% Triton X-100 in PBS. The cells were then incubated at 4°C with the primary antibody anti-CFTR (clone CF3, Abcam) and/or anti-GRASP55 (Proteintech, 10598-1-AP). After extensive washing with PBS, the slides were incubated at room temperature for 60 minutes with goat anti-mouse antibody to CFTR followed by Alexa Fluor 555 (BioLegend, 405324) and Alexa Fluor 488 anti-phalloidin (Thermo Fisher Scientific, A12379) for F-actin labeling. Cell were pretreated with SiRNA for GRASP55 or 10 μg/mL brefeldin A (Sigma-Aldrich) for 24 or 6 hours, respectively, at 37°C before immunostaining. For IHC, the tissues were removed and fixed in 10% phosphate-buffered formalin, embedded in paraffin, and sectioned at 5 μm. Sections were then rehydrated, and after antigen retrieval in citrate buffer (10 mM, pH 6.0), sections were fixed in 4% formaldehyde for 40 minutes at room temperature and permeabilized in blocking buffer containing 5% FBS, 3% BSA, and 0.5% Triton X-100 in PBS. The sections were incubated at 4°C overnight with anti-CFTR (CF3, Abcam) antibody followed by biotinylated secondary antibodies. DAPI and hematoxylin were used to counterstain nuclei. All images were acquired using a BX51 fluorescence microscope (Olympus) with ×20 and ×100 objectives using analySIS image-processing software (Olympus).

### Functional analysis of CFTR.

Patch-clamp recordings were performed from p.Phe508del-CFTR–transfected FRT cells treated with 10 μg/mL anakinra, 3 μM VX-809 (Aurogene), or vehicle for 4 hours at 37°C. Pipettes with resistance of 2–4 MΩ were pulled from borosilicate glass capillary tubing (1B150F-3; World Precision Instruments), using a 5-step horizontal puller from Sutter Instrument. Cells were stimulated with forskolin (10 μM), genistein (30 μM), or 10 μM VX-770 (Aurogene). Macroscopic CFTR currents were recorded in whole-cell dialyzed configuration using an EPC-10 patch-clamp amplifier (HEKA Elektronik). I–V relationships were built by clamping the membrane potential of FRT-cells at –40 mV and by delivering ramps from –100 mV to 50 mV. The pipette solution contained (mM): 113 L-aspartic acid, 113 CsOH, 27 CsCl, 1 NaCl, 1 MgCl_2_, 1 EGTA, and 10 TES (pH 7.2). MgATP (3 mM) was added just before patch-clamp experiments were started. The external solution contained the following (mM): 145 NaCl, 4 CsCl, 1 CaCl_2_, 10 glucose, and 10 TES (pH 7.4). Results were analyzed with FITMASTER software (HEKA Elektronik) and Microcal Origin 8.0. A Kolmogorov-Smirnov normality test turned out to be nonsignificant. The variance was similar in the groups being compared. We considered all *P* values of 0.05 or less to be significant. Ussing chamber experiments were performed as described (58). Chamber solution was buffered by bubbling with a mixture of 95% O_2_ and 5% CO_2_. HBE cells were short-circuited using Ag/AgCl agar electrodes. Short-circuit current and resistance were acquired or calculated using the VCC-600 transepithelial clamp from Physiologic Instruments and Acquire & Analyze 2.3 software for data acquisition as previously described (58). A basolateral-to-apical chloride gradient was established by replacing NaCl with sodium gluconate in the apical (luminal) compartment to create a driving force for CFTR-dependent Cl^−^ secretion. CFTR channels present at the apical surface of the epithelium (lumen side of the tissue) were activated. Stimulations with forskolin, CFTR inhibitor 172, and amiloride were performed as described (58).

### Trp metabolites quantification by LC high-resolution mass spectrometry.

The frozen cell samples (3 × 10^6^) were thawed at 4°C and spiked with a suitable concentration of labeled internal standards (kynurenic acid-d5 and tryptophan-d5) in 2 mL tubes. After the addition of 250 μL of a solution urea/Tris HCl (pH 7.4), the tubes were vortexed for 15 seconds and sonicated for 15 minutes. Then, 750 μL of acetonitrile was added and samples vortexed and centrifuged (16,800*g*, 15 minutes). The supernatants were transferred into a 2 mL tube. After evaporation under nitrogen stream (40°C), the samples were resuspended in 100 μL of a mixture H_2_O/MeOH 95:5 (v/v), shaken, and then injected in the LC high-resolution mass spectrometer (LC-HRMS) system. Chromatographic separation was performed on the Ultimate 3000 HPLC system (Thermo Fisher Scientific). Stock solutions (100 mg/mL) were prepared by dissolving each standard in H_2_O/MeOH, 90:10 (v/v), except kynurenic acid and kynurenic acid-d5, which were dissolved in DMSO. Analytes were separated on a XSelect HSS T3 column (100 × 2.1 mm, 1.7 μm, Waters Corporation) connected with a guard column (2.1 × 5 mm, Waters Corporation). LC eluents A and B were water and acetonitrile (ACN), both containing 0.1% (v/v) formic acid. From 0 to 9 minutes, the percentage of eluent B increased from 0% to 15% and from 9 to 9.9 minutes to 100%. This condition was maintained for 1 minute and then the system returned to 0% B (0.1 minute) and it was reequilibrated for 2.5 minutes (run time: 13.5 minutes). The column and autosampler temperatures were kept at 40°C and 16°C, respectively. The flow rate was 0.30 mL min^−1^ and injection volume 10 μL. The mass spectrometer Q-Exactive Plus (Thermo Fisher Scientific) was equipped with a heated electrospray ionization (HESI-II) source. The HESI-II temperature was set at 350°C, the capillary temperature at 300 °C, and the electrospray voltage at 4.00 kV (positive mode). Sheath and auxiliary gas were 40 and 15 arbitrary units. The mass analyzer was controlled by Xcalibur 3.0 software (Thermo Fisher Scientific). The acquisition was achieved in full scan/dd-MS^2^ mode. Quantitative analysis was performed using full scan data. The mass scan range was *m/z* 100 to 500. The data were acquired at a resolving power of 70000 full-width at half-maximum (FWHM) (*m/z* 200). Automatic gain control (AGC) was set at 1 × 10^6^ ions for a maximum injection time of 320 ms. The precursor ions, filtered by the quadrupole (isolation window equal to *m/z* 1.0), were fragmented with stepped normalized collision energies at 120, 30, and 50 eV. An inclusion list for dd-MS^2^ experiments was used, including for each analyte the *m/z* of precursor ion and expected retention time (±1 minute). A resolving power of 17,500 FWHM (*m/z* 200) was used for dd-MS^2^ experiments with AGC target set at 5 × 10^5^ ions and injection time at 80 ms. Kynurenine and its metabolites were quantified by applying isotopic dilution methodology.

### Senescence assay.

MEF cells from passage 4 to 7 were seeded at 1.5 × 10^5^ cells per well in 24-well plates and exposed to 10 μg/mL anakinra at 37°C. Triplicate dishes were counted at each passage. MEF cells at passage 6 were subjected to immunofluorescence staining by incubation, after fixation and permeabilization, with the rat monoclonal anti-p19Arf (Merck KGaA, Calbiochem, CB1012) as a marker for senescence, followed by the secondary antibody Alexa Fluor 555 Texas red–conjugated anti-rat IgG (Invitrogen, A-21434). DAPI was used to detect nuclei.

### ELISA.

Cytokine content was determined in lung homogenates by using specific ELISA kits according to the manufacturers’ instructions (BioLegend, eBioscience Inc., and R&D Systems). Kynurenine production was evaluated in cell supernatants (LDN). The cytokine and kynurenine concentrations were expressed as pg/mL or nmol/L, respectively.

### RT-PCR.

RT-PCR was performed using the CFX96 Touch Real-Time PCR detection system and iTaq Universal SYBR Green Supermix (Bio-Rad). Lung and cells were lysed and total RNA was isolated with TRIzol Reagent (Thermo Fisher Scientific), and cDNA was synthesized using the PrimeScript RT Reagent Kit with gDNA Eraser (Takara), according to the manufacturer’s instructions. Each data point was examined for integrity by analysis of the amplification plot. The thermal profile for SYBR Green RT-PCR was at 95°C for 3 minutes followed by 40 cycles of denaturation for 30 seconds at 95°C and an annealing/extension step of 30 seconds at 60°C. Amplification efficiencies were validated and normalized against *Gapdh*. The mouse primers (5′–3′) were as follows: *Gapdh*: TCGTCCCGTAGACAAAATGG and TTGAGGTCARGAAGGGGTC; *Ahrr*: AGAGGGTTCCCCGTGCAG and ACTCACCACCAGAGCGAAGC; *Cyp1α1*: ACAGTGATTGGCAGAGATCG and GAAGGGGACGAAGGATGAAT *Cyp1b1*: TTCTCCAGCTTTTTGCCTGT and TAATGAAGCCGTCCTTGTCC; *Gpx7*: CCCATTCCTGAACCTTTCAA and GCACACGAAACCCCTGTACT; *Ido1*: CATGACATACGAGAACATGGAC and GACAGATATATGCGGAGAACG; *Ido2*: CATGGCGCTGGCCGCTATCA and TTAAGGCCGGGCACTGCTGC; *Nox4*: GAAGATTTGCCTGGAAGAACC and AGGTTTGTTGCTCCTGATGC*; p22^phox^:* ACCTGACCGCTGTGGTGAA and GTGGAGGACAGCCCGGA; *Sod3*: CCTAGCAGACAGGCTTGACC and GTCGTCCTAGCTCCATCCAG; *Tdo2*: ATGAGTGGGTGCCCGTTTG and GGCTCTGTTTACACCAGTTTGAG.

### QuantiGene Plex gene expression assay.

Total RNA from experiment was extracted with TRIzol reagent (Thermo Fisher Scientific) according to the manufacturer’s instructions. The total RNA was qualified and quantified by the Tecan Infinite 200 microplate reader (Tecan Group Ltd) following the instrument’s protocols. The RNA was diluted using nuclease-free water. The sample input range was 250 ng/well. All QuantiGene probe sets are available from Thermo Fisher Scientific, and the individual accession numbers and probe set regions are summarized in Supplemental Table 4. The QuantiGene assays were performed as indicated in the manufacturer’s protocol. Plates were read using Magpix (Luminex). Data analysis was performed by normalizing the signals obtained for the genes of interest to the geometric mean of the reference gene signals (Polr2a, Tfrc, and Hprt).

### Statistics.

GraphPad Prism software 6.01 was used for the analysis. Statistical significance was calculated by 1 or 2-way ANOVA (Tukey’s or Bonferroni’s post hoc test) for multiple comparisons and by a 2-tailed Student’s *t* test for single comparisons. The distribution of levels tested by Kolmogorov-Smirnov normality test turned out to be nonsignificant. The variance was similar in the groups being compared. We considered all *P* values of 0.05 or less to be significant.

The colocalization program ImageJ (59) with the JACoP plug-in was used to quantify the degree of overlap by calculating the colocalization coefficients (Pearson’s correlation coefficient, overlap coefficient according to Manders, and the overlap coefficients as reported in Supplemental Figure 8).

### Study approval.

Mouse experiments were performed according to Italian Approved Animal Welfare authorization 360/2015-PR and Legislative Decree 26/2014 regarding the animal license approved by the Italian Ministry of Health (Rome) lasting for 5 years (2015–2020). 

## Author contributions

FLVDV and LR conceived and designed experiments. ADL, M. Pariano, RGI, MB, and GR performed in vivo experiments. M. Pariano, VO, FDO, CS, M. Puccetti, MMB, SG, MC, and MADF contributed to Western blot and IP, ELISA, RT-PCR, immunofluorescence, and IHC experiments. VRV and LM performed Ussing chamber experiments. DP and GS analyzed RNA-Seq data. SP performed ChIP experiments. BC performed limited proteolysis experiments. FF, MG, and GM performed the luciferase reporter experiments; RG and CB performed LC-HRMS; LS performed patch clamp experiments. LR wrote the paper. FLVDV, VNT, CC, and CAD edited the paper. LR supervised the work.

## Supplementary Material

Supplemental data

## Version 1 11/30/2021

In-Press Preview

## Version 2 01/18/2022

Electronic publication
