# Supplementary material for: Anakinra restores cellular proteostasis by coupling mitochondrial redox balance to autophagy
Source: J Clin Invest. 2022 Jan 18;132(2):e144983. doi: 10.1172/JCI144983 (PMC8759782; doi:10.1172/JCI144983)

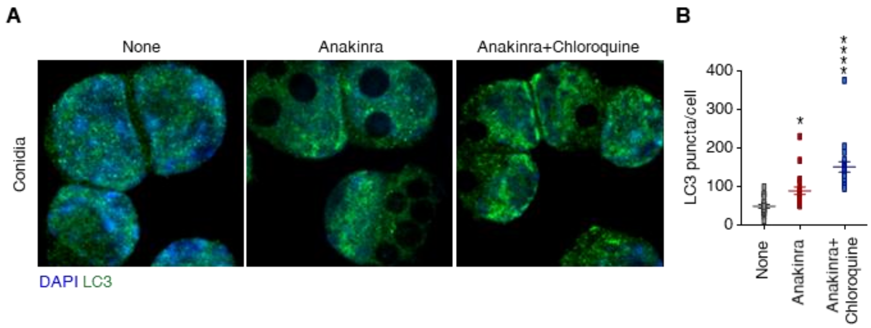

Supplemental Figure 1. Anakinra-induced autophagy in the presence of chloroquine. (**A, B**) LC3 staining of RAW 264.7 cells exposed to *A. fumigatus* conidia and treated with 10μg/ml anakinra with and without 100 μM Chloroquine for 4 h. (**B**) Mean percentage of LC3 puncta/cell (n = 20). DAPI was used to detect nuclei. Data represent the mean ± SEM of one representative out of three independent experiments. *P <0.05, ****P < 0.0001, treated *vs.* untreated (None). One-way ANOVA, Bonferroni post-hoc test.

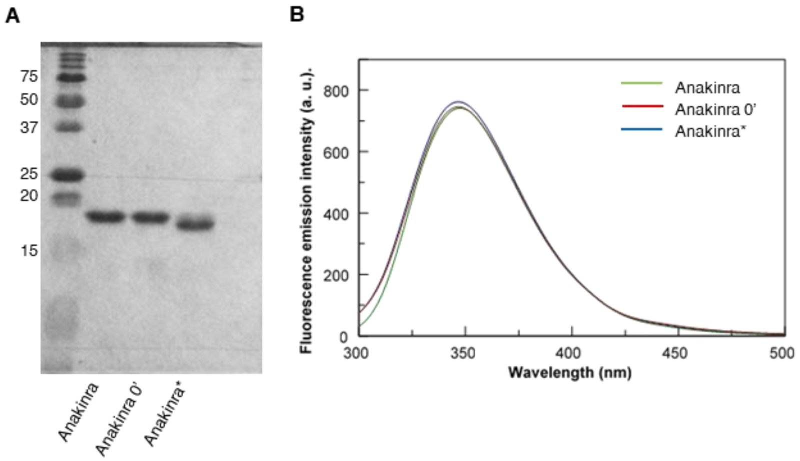

Supplemental Figure 2. Limited proteolysis of anakinra by proteinase K. (**A**) Anakinra was treated with proteinase K for 60’ and the reaction was blocked by the addition of PMSF (anakinra*) before separation in SDS-PAGE. As control, full-length anakinra and anakinra treated with proteinase K and PMSF from the beginning (anakinra 0) were loaded in parallel. (**B**) Intrinsic fluorescence emission spectra of 5 μM full-length anakinra (green line) and upon treatment with proteinase K in the presence of PMSF from the beginning (red line) or after 60' (blue line) in PBS at 25 °C. Excitation wavelength 280 nm. Data are representative of at least three independent experiments.

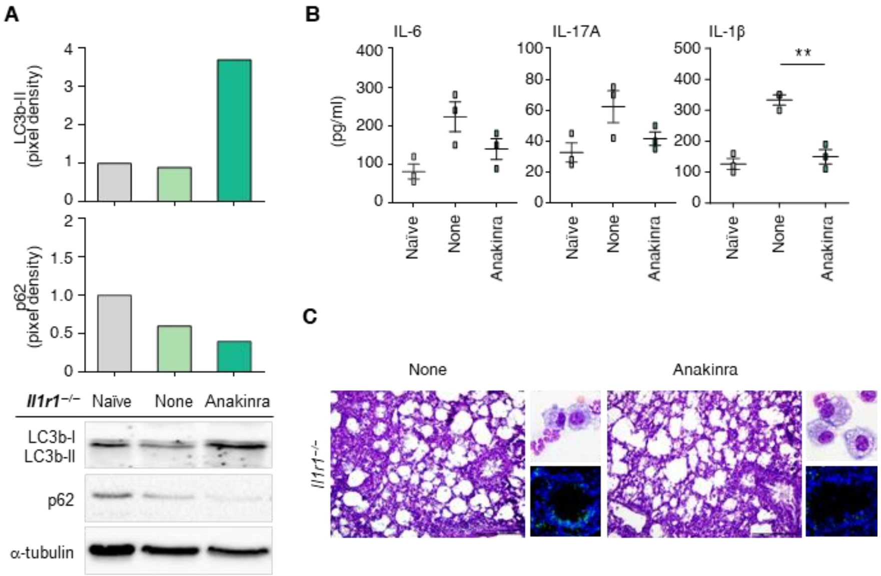

Supplemental Figure 3. Anakinra induces autophagy and limits inflammation in *Il1r1*$^{-/-}$ mice with *Pseudomonas aeruginosa* infection. *Il1r1*$^{-/-}$ mice were infected i.n with live *P. aeruginosa* cells, treated with 10 mg/kg anakinra i.p. for 6 consecutive days. Mice were sacrificed at 7 days post infection and assessed for immunoblotting of LC3b and p62 (**A**), cytokine production (ELISA) in lung homogenates (**B**) and lung histology [PAS staining], BAL morphology and TUNEL staining (**C**). Scale bar 200 μm. Data are representative of one out of two independent experiments. Naïve, uninfected mice. None, untreated mice.

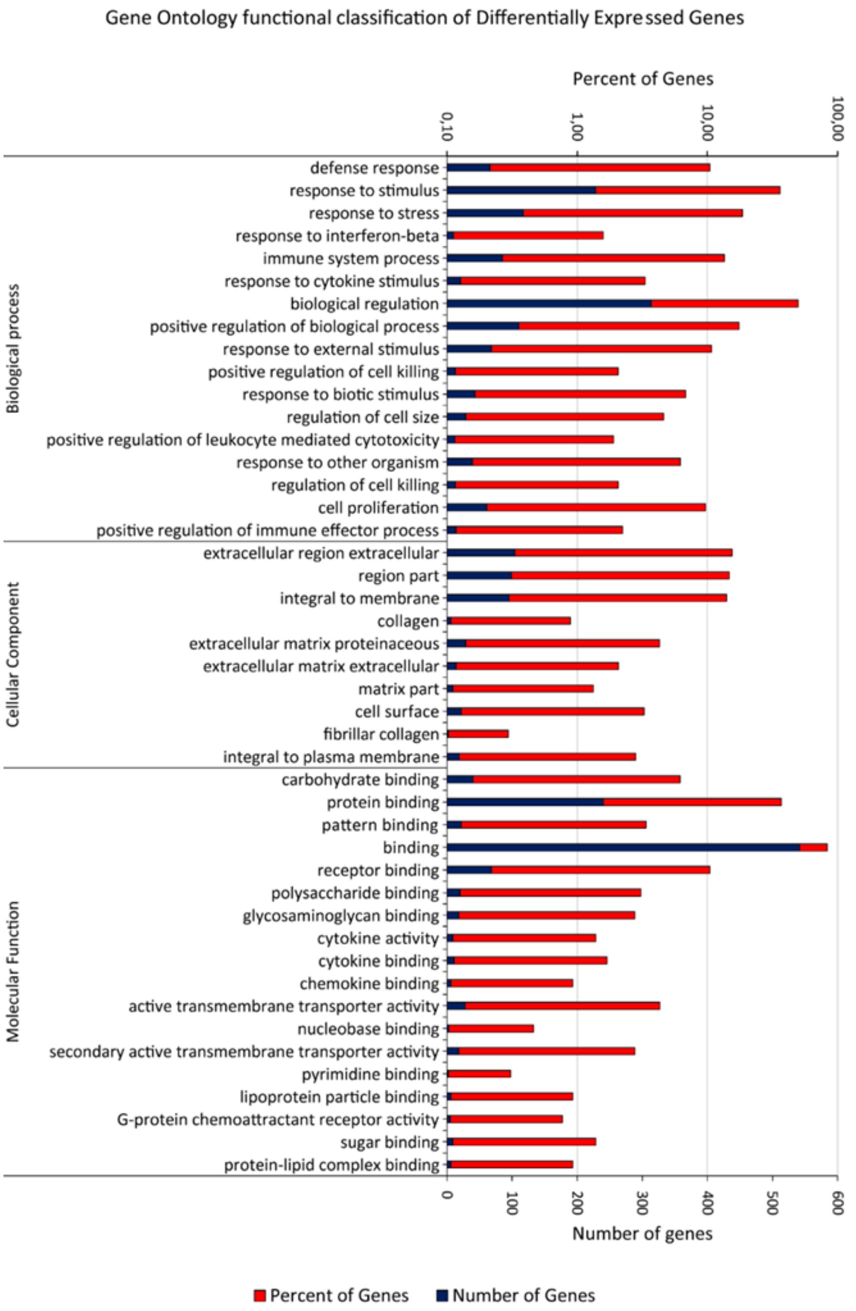

Supplemental Figure 4. Gene ontology functional classification analysis of differentially expressed genes. Genes were annotated by Gene Ontology terms that are summarized in three main categories of biological process, molecular function and cellular component.

Figure 2E. MITOSOX

Min	None *vs* Anakinra
0	n.s.
5	n.s.
10	n.s.
15	n.s.
20	n.s.

Figure 2F. DHE

Min	None *vs* Anakinra
0	n.s.
5	n.s.
10	n.s.
15	n.s.
20	n.s.

Figure 2G. DHR

Min	None *vs* Anakinra
0	n.s.
15	n.s.
30	n.s.
45	****
60	****

Figure 6E. DHR

Min	None *vs* Anakinra	Anakinra *vs* Anakinra + MITO
0	n.s.	n.s.
20	n.s.	n.s.
40	**	n.s.
60	**	n.s.

Figure 7B. AMPLEX RED

Min	None *vs* Anakinra
0	n.s.
15	n.s.
30	n.s.
45	*
60	**

Figure S7A. DHR – C57BL/6

Min	None *vs* Anakinra	Anakinra *vs* Anakinra + MITO
0	n.s.	n.s.
15	*	n.s.
30	****	****
45	****	****
60	****	***

Figure S7A. DHR - *Il1r1*$^{-/-}$

Min	None *vs* Anakinra	Anakinra *vs* Anakinra + MITO
0	n.s.	n.s.
15	**	*
30	****	****
45	****	****
60	****	****

Supplemental Figure 5. P values of the relative figures.

Figure 2I	None	Anakinra	Starvation
Pearson's coefficient	0.895	0.815	0.845
Overlap coefficient	0.936	0.844	0.976
Manders' coefficient_DCF-DA/Mitotraker	0.995	0.815	0.999
Manders' coefficient_Mitotraker/DCF-DA	0.993	1.0	1.0

Figure 4D	C57BL/6	*Il1r1*$^{-/-}$
Pearson's coefficient	0.810	0.858
Overlap coefficient	0.825	0.885
Manders' coefficient_Anakinra/AhR	0.980	0.903
Manders' coefficient_AhR/Anakinra	0.940	0.982

Figure 4E	MEF *Il1r1*$^{+/+}$ cells	MEF *Il1r1*$^{-/-}$ cells
Pearson's coefficient	0.245	0.151
Overlap coefficient	0.339	0.288
Manders' coefficient_Anakinra/AhR	0.682	0.898
Manders' coefficient_AhR/Anakinra	0.373	0.530

Figure 8B	Untreated		Brefeldin A		SiGRASP55	
	None	Anakinra	None	Anakinra	None	Anakinra
Pearson's coefficient	0.885	0.803	0.443	0.835	0.272	0.632
Overlap coefficient	0.927	0.882	0.416	0.906	0.557	0.679
Manders' coefficient_GRASP55/CFTR	0.997	0.999	0.999	1.0	0.999	0.954
Manders' coefficient_CFTR/GRASP55	0.963	0.931	0.108	0.925	0.925	0.794

Supplemental Figure 6. Co-localization coefficients of the relative figures.

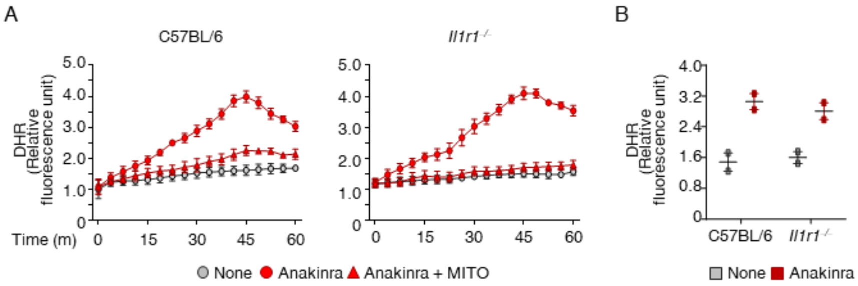

Supplemental Figure 7. Mitochondrial H_2O_2 production in the absence of IL-1R1. (**A**) DHR fluorescence in ex-vivo purified alveolar macrophages exposed to *Aspergillus* conidia and anakinra and (**B**) total lung cells from infected mice (7 days after the infection) treated with anakinra as in legend to Figure 1. Data are the means ± SEM of one representative out of two independent experiments. For P values in (**A**), see Supplementary Figure 7.

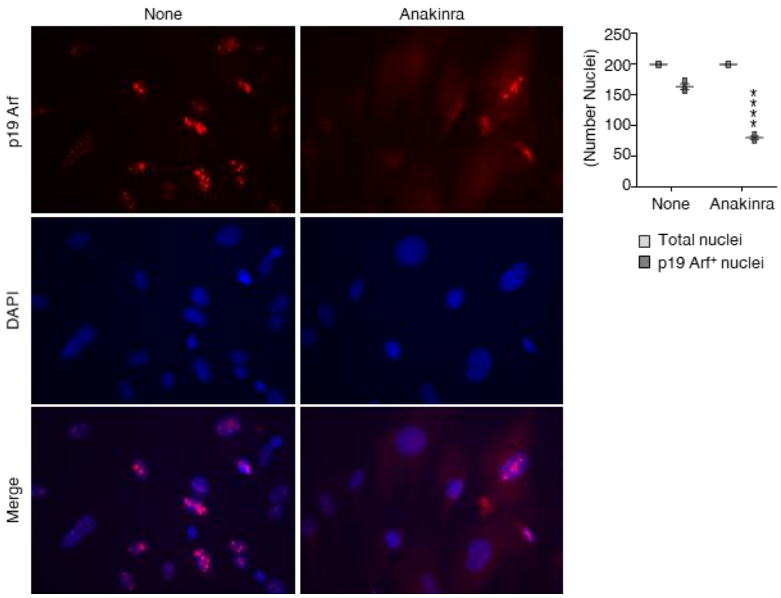

Supplemental Figure 8. Anakinra retards senescence. MEF cells at passage 6 were exposed to anakinra and subjected to immunofluorescence analysis by incubation with anti-p19Arf followed by the secondary antibody Alexa Fluor555 Texas-Red-conjugated anti rat immunoglobulin. DAPI was used to detect nuclei. The graph is a schematic representation of the counts of the total nuclei and p19 Arf^{+} nuclei in untreated and treated cells. Data represent the mean ± SEM of three determinations. ****P <0.0001. Two-way ANOVA, Bonferroni post-hoc test.

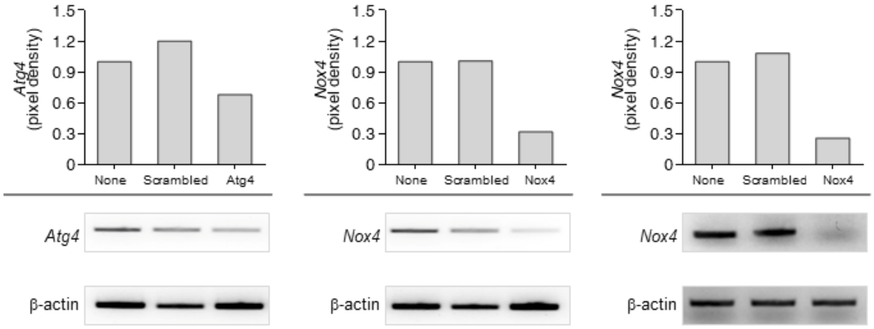

Supplemental Figure 9. Expression of *Atg4a* and *Nox4* by RT-PCR in RAW 264.7 cells exposed to specific SiRNA 24h before. On the right, expression of *Nox4* in ex-vivo lung cells from infected *Il1r1*$^{-/-}$ mice treated with SiNox4 as in legend to Figure 3A.

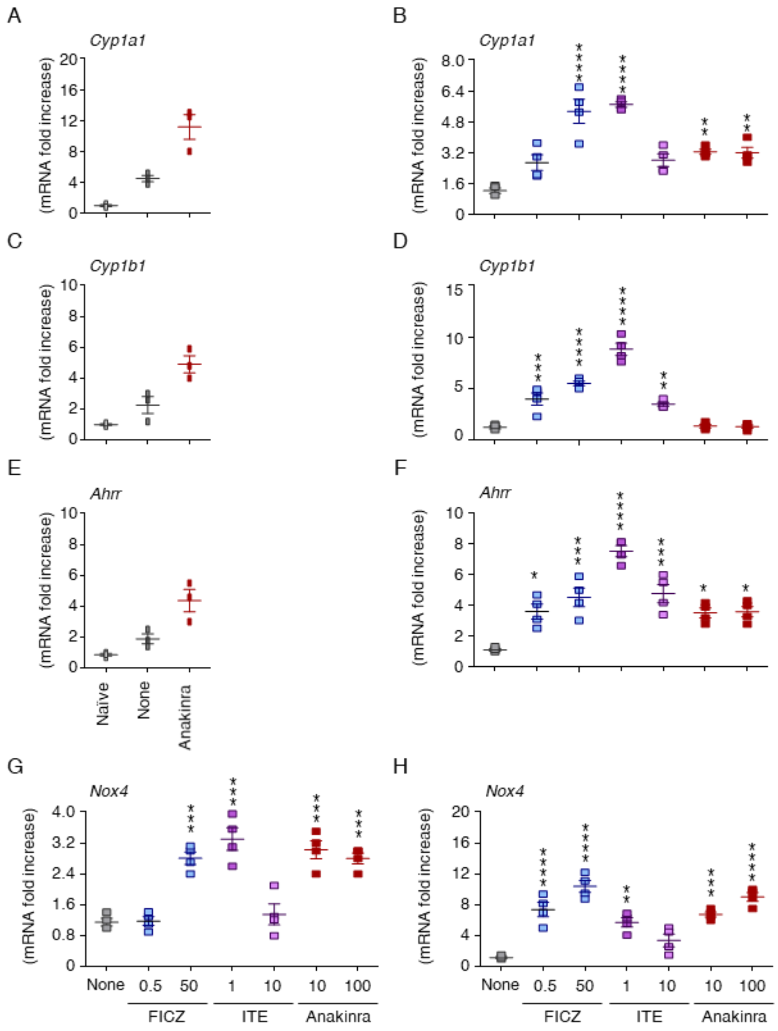

Supplemental Figure 10. Anakinra activates AhR in the absence of IL-1R1. Expression of the AhR signature genes *Cyp1a1*, *Cyp1b1*, *Ahrr* and *Nox4* by RT-PCR in (**A, C, E**) *Il1r1*$^{-/-}$ mice infected with live *Aspergillus* conidia (6-8 mice/group) and treated with 10 mg/kg anakinra intraperitoneally for 6 consecutive days before the assay. Data are technical replicates of one representative out of two independent experiments. (**B, D, F**) ex-vivo lung cells from naïve *Il1r1*$^{-/-}$ mice (n = 3-4 independent samples) and (**G, H**) RAW 264.7 cells (n = 3-4 independent samples) exposed to anakinra (10 and 100 μg/ml) or 6-formylindolo[3,2-326 b]carbazole (FICZ) (0.5 and 50 nM) or ITE (1 and 10 μM) for 2 (**G**) or 4 (**H**) h at 37°C in vitro. * P <0.05, **P <0.01, ***P <0.001 and ****P <0.0001, anakinra- or AhR ligand-treated *vs*. untreated (None) cells or mice. One-way ANOVA, Bonferroni post-hoc test.

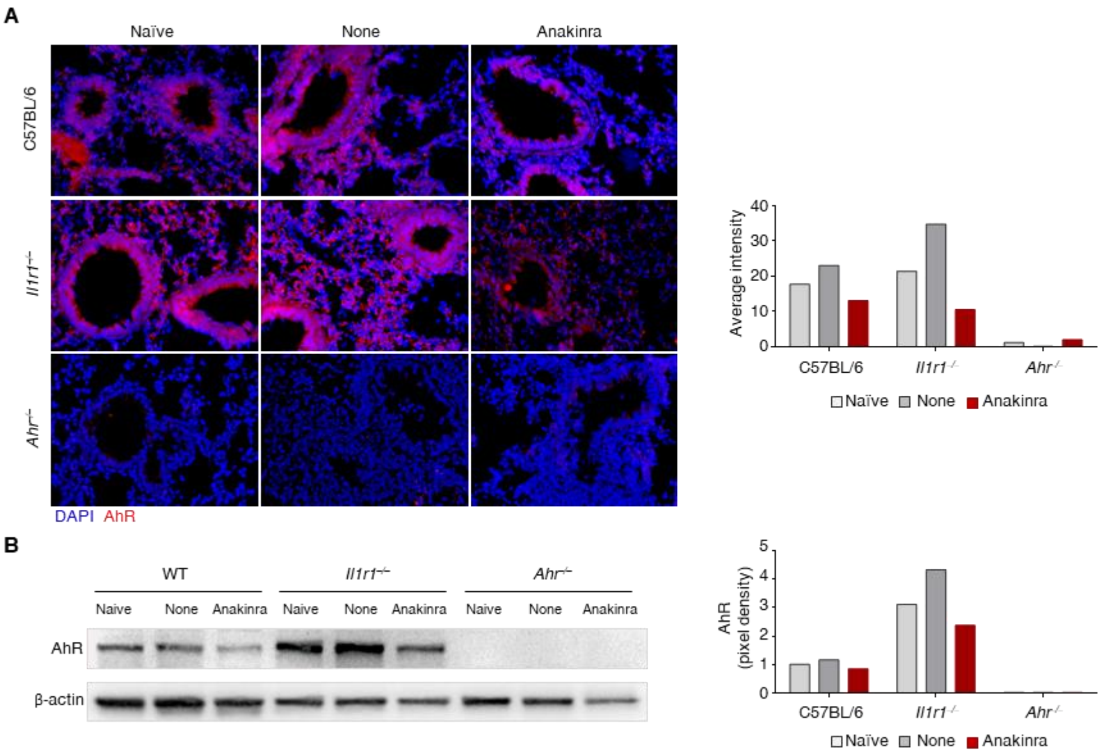

Supplemental Figure 11. AhR expression is increased in the relative absence of IL1R1. AhR protein expression by immunofluorescence staining (**A**) and western blotting (**B**) of lungs from C57BL/6 and *Il1r1*$^{-/-}$ either naïve, or infected with *A. fumigatus* (None) and treated with anakinra, as detailed in Methods. Assays were done a week after the infection, Both immunofluorescence staining and western blotting revealed that AhR expression was apparently higher in *Il1r1*$^{-/-}$ than control mice, particularly in infection and was reduced upon its activaction by anakinra, a finding consistent with the observation that AhR is rapidly degraded following activaction (*Davarinos NA and Pollenz RS. Aryl hydrocarbon receptor imported into the nucleus following ligand binding is rapidly degraded via the cytoplasmatic proteasome following nuclear export. JBC, 274:28708-15, 1999*). The average intensity was obtained by quantification of the corresponding average immunofluorescence intensities of AhR by using Image J software on 142x142 pixel area. (*Collins, T.J. ImageJ for microscopy. Biotechniques 43: 25-30, 2007*). AhR$^{-/-}$ mice were used as antibody control.

Supplemental Table 1. RNA-Seq data in purified C57BL/6 (A) and *Il1r1*$^{-/-}$(B) alveolar macrophages exposed to anakinra in vitro.

Names	Total	Elements
**A∩B**	110	Hmga2-ps1 Zfp383 Retnla Lnpep Zbtb16 Cish Rs5-8s1 Nr4a3 Pfkl Csf3 Tnfaip6 Smtn Slc16a3 Tubb1 Lyst Narf Tmem254a Fcna Ptafr Adgb Lamc2 Zfp433 Snhg5 Hist2h2aa1 Setd1b H2-Q8 Slc2a4rg-ps Sh2b2 Siglec5 Npr3 Rorb Shb Eif3j1 Gdap10 Ptges Pet100 Atp2b4 Slc6a14 Itgax Kcnn3 Rnu2-10 Fam129a Hhip Zfp292 Irs2 Saa3 Plac9a Atg7 Ppp1r14a Gpnmb Ddit4 Prg4 Pgap1 Ppbp Osmr Gfi1 Flnc Havcr2 Ago-02 Hsph1 Mmp3 Fosl1 Socs1 Rhoc Colq Rest Bbip1 Fam83g Pou2f1 Col5a2 Wdr96 Tfpi2 Ppp1r3b Gimap3 Cldn4 Prkcg Tpi1 Pqlc1 Rab44 Pvr Creb3l1 Selp Ch25h Chac1 Rel Itga2b Pgam1 Mif Gpr4 Igtp Slc2a1 Spata5l1 Egln3 Oaf Mast4 Cdo1 Dynlt1a Serpinb2 Hjurp Alox15 Crip1 Mat2a Srsf4 Fam124a Tuba1c Armcx4 Acta1 Bcar1 Rnd1 Il12b
**A**	758	Syt13 Mtmr10 Wdr81 Lyve1 Btbd3 Kcnd3 Angptl4 C1ra Gstm2 Cyp2s1 Mmp2 Mcam Islr Cyp7b1 Gsta3 Spock2 Cmbl Itgb7 Ikbke Myo1g Car3 Cyth1 Kirrel Parp1 Slc36a2 Adamts9 Med12 Sept4 Rapgef5 Rbp7 Gpr174 Pcdhgc3 Ctps Plcxd2 Pdgfrb Loxl1 Ndrg1 Myo1b Tlr9 Gltscr1 Spon1 Lrat Wt1 Synpo Kif21b Lrp2 Plxdc2 Xpo1 Mamdc2 Egfr Cd22 C1qa Lgals2 Mpzl1 Klhl21 Tubb6 Ctdspl Sln Ppp1r2 Col4a2 Pde3b Nid1 Mxra7 Ptpn22 Fkbp9 Il1r1 Plod2 Ocln Plcg2 Cd19 Ppap2b Smox Rgs1 Mst1r Cdh11 Rtn4 Stk10 Cacna1d Rhou Hbegf Agpat9 Hspg2 Tet2 Slit2 Zmiz2 Cxcl13 Mfap2 Cx3cr1 Dock3 Ncs1 Timp1 Olfml3 Ly6c1 Sash3 Tmem35 Rhobtb2 Nnmt Aspa Espl1 Krt8 Xcl1 B3gnt7 Mrc2 Tbx4 Bcl6b Treml1 N4bp2l1 Medag Ninl Cd63 Pla2g7 Fam101b Mdm4 Adra1b Nipal1 Tmc8 Selm Col6a1 C1qtnf1 Tcf21 Card6 Steap1 Cldn5 Ciita Tmem194b Angpt1 Ffar2 Tjp2 B3gnt5 Cytip Irak1bp1 Pde7a Nox4 B4galnt1 Ctgf Gria1 Tfpi Tead1 Tnip3 Glycam1 Tnks1bp1 Spata13 Ero1lb Ddah2 Plau Apln Rspo1 Lef1 Fyb Dpt Kcne4 Pls3 AI504432 Sfrp1 Runx2 Rdh10 Prdm1 Zfp939 Zfp36l2 Gas1 Slc10a6 Pmp22 Il7r Ecm1 Matk Procr Klrb1b Ap1g2 Pdzd7 Samd4 Ptpn14 Pparg Itga4 Rasal3 Il1f9 Eno1 Sema4d Rarres2 Prkg2 Ces1d C1qc Serpina3n Stard13 Crispld2 Bank1 Lax1 Adck3 Ms4a6c Cilp2 Rhov Slc6a8 Fcrl1 Rasa4 BC051226 Upk3b Ddr1 Ebf1 Efemp1 Papss2 Adamts4 Pou2f2 Kctd11 St8sia6 Adh1 Cd28 Tns1 Faim3 Plekha2 Sh2d2a Htra1 Rbfox2 F2r Rasl12 Pkhd1l1 Ube2v2 Ccdc88c Myo1f Ddr2 Sphk1 Plbd1 Tmem119 Adamts14 Mfge8 Acan Atp1a2 Scgb3a1 Slc5a5 Serpinb10 Cdkn1c Hvcn1 Eomes Tinagl1 Col18a1 Col4a5 Fam84a Slc35f2 Aebp1 Ctsl Slamf7 Dock8 Igfbp3 H2-Ob Basp1 Btla Gpr132 BC035044 Clnk Ngf Rbpj Boc Fam198b Inpp5d Gucy1b3 Rcn1 Klhl6 Dcbld2 Sema4c Spock3 Arhgap4 Layn Figf Slc25a25 Gpr35 Wfdc1 Arhgef17 Zfp53 Unc5b Pdzd2 Fibin Spib Neo1 Rapgef3 Fbln5 Itga8 Crk Cfh Mras Dennd4b Il33 Col5a1 Il1rl1 Syk Prf1 Fndc9 Cd79b Myrf Rasip1 Colec12 Cyp26b1 Sox6 Robo4 Pdpn Aim2 Pcolce Sema6b Map3k6 Plcl2 Rcn3 Vgll3 Atf5 Dmpk Cyfip2 Dok5 Gabarapl1 Il6 Tnfsf14 Wwc2 Slc7a10 Fam20c Robo2 Eif2ak3 Iigp1 Slc9a7 Dmwd S100a16 Naip5 Cacna2d1 Gpr126 Acta2 C7 Klhl24 Thada Smoc2 Cnn3 Spry4 Des Tubb3 Gpm6a Scnn1a Rcan1 H2-Q6 Egflam Kcnk3 Ikzf3 Cwc22 Myef2 Cxadr Gcnt1 Palm2 Slc35f5 Rhof Ptx3 Abcc1 Scube2 Klhdc8b Ptk2b Col23a1 Wwtr1 Endou Emilin2 Cdk5r1 Traf3ip3 Bend4 Tagln Upk1b Pnpla7 Tnfrsf10b Tnfrsf13c Il21r Myh10 Alox12 Kbtbd11 Fam107a Has1 Lrrn4 Fam180a Pla2r1 Fstl1 Gata3 Wnt2 Btbd16 Ehf Svep1 Arhgap28 Trip10 Krt17 Ltbp1 Ndrg2 Xylt1 Ppp4r4 Pard6b Tuba1a Sgce Plat Gnai1 Jph1 Crtap Fibcd1 Fendrr Atp8b1 C1qb Col16a1 Ptger4 Igfbp7 Zbtb7c Sod3 Col4a3 Prx Adamts15 Cyp2b10 H2-DMb2 Stbd1 Slc11a1 Abca8b Tubb4a Il11 Cebpd Postn Scarf2 Cald1 Rhoh Tslp Pdcd4 H6pd N4bp2 Kcna3 Pgm5 Plxna2 Rbms3 Scn3a Pik3ap1 Siglecg Prex2 Vps37b Chst3 Adamtsl2 Ifnb1 Mfap5 Ptgis Rusc2 Slc38a5 Adamts8 Grap2 Akna C4b Fgfr4 Rasgrp1 Bach2 Foxc2 Cldn15 Osgin2 Slc2a10 Fscn1 Rpl36 Fam101a Map3k1 Aplp1 Nrg2 Tmem176b Mdn1 Leprel4 Nebl Nt5dc2 Cnr2 Col6a2 Spon2 Col13a1 Ndst1 Aldh1b1 Fcer2a Cdkn1b Rlf B3gntl1 Gbp6 Phlda3 Dll1 Tgfb2 Fgfr1 Treml4 Tlr1 Cp Pla2g2d Carhsp1 Anxa8 Irf4 Fmod Mxra8 Tmem151a Pdk4 Rhoj Kcnh4 Tspan4 Slc43a2 Nkain4 Eda2r Astn2 Sele Sulf1 F10 Hist1h1e Timp2 Lox Tctn2 Cd300e Ifit1 Lamb1 Trim9 Psg23 Dtx1 Prss23 Hspb8 Arhgap26 Csf2 Adam8 Cpe Rag1 Dnahc7a AU021092 Hecw2 Yap1 Prkcb Pkd2 C2 Krt7 Dennd2a Amz1 Rbp1 Itpkc Ifitm3 Irgm1 Ubtd2 Dntt Nbl1 Mdfi Gpr183 Nme7 Lpar1 Tnnt2 Gja1 Cxcl14 Cxcl5 Dpep1 Cep112 Fermt2 Fam150a Olr1 Agap2 P2ry10 Cd72 Fjx1 Tgfbi Pkdcc Darc Mapk4 Ms4a1 Clec7a Ildr2 Klra3 Mical1 Ptk7 Lix1l Tacc2 Zc3h12c Reg3g Apold1 Sprr1a Stk17b Ablim3 Shroom3 Lzts2 Serping1 Srf Loxl2 Clu Hspb1 Slfn4 Igf2bp2 Pced1b Mreg Gpr133 P4ha2 Slc2a3 Ugdh AB124611 Amph Tmem45a Hsd11b1 Slc7a11 Gprc5a Kcnip2 Tpm1 Nfatc3 Col6a3 Rhbdf1 Spag11b Trim16 Pik3ip1 Wisp2 Prex1 Ppp1r16b Aass Arl5c Gucy1a3 Snx5 Evi2b Jarid2 Runx3 Trim65 Rasgef1b Epha2 Acvr1 Ier3 Aldh1a2 Mef2c C1s Prelp Gpd2 Map3k14 Agtr1a Dusp8 Pgm1 Sesn3 Fxyd1 Cpne5 Zfp318 Ido1 Tsku Nppa Grb10 Serpine2 Bmper Spsb1 Srpk3 Adarb1 Tnnc2 Aspm Flrt3 Dock2 Nes Ror2 Fam69a Fam169b Klf15 Cdr2 Lmo7 Ms4a4d Sbsn Faim2 Igj Fbln2 Stxbp3b Spo11 Rasa3 Fras1 Gpr84 Ager Vipr2 Hist1h1c Cttn Ifi202b Ccne1 Ccdc80 Serpina3h S1pr3 Sephs2 Htra3 Inpp4b Gpcpd1 Pde2a Gpm6b Bcl2l11 Lhfp Mll5 Tnfsf9 Erc2 Nlgn2 Stc1 Pawr Il18rap Rnd3 Mettl18 Tmem204 Ism1 Slc15a4 Pxdc1 Vtcn1 Lama2 Steap4 Cyp4f18 Kcna1 Nuak1 Serpinh1 Cpq Slurp1 Ptprd Reck Has2 Psd3 Pax5 Kazn BC022687 Notch3 Lipg Smo Pnp Wdfy4 Zfp608 Numbl Dgka Fblim1 Slc16a1 Col6a5 Dnajb9 Prob1 Bmpr1a Leprel2 Itgb3 Ltbp2 Mir5107 Mettl7a1 Ipcef1 Traf1 Zbtb18 Vcl Pcolce2 Nckap1l Pvrl3 Tnc Malt1 Sdc2 Phldb1 Nkg7 Krt18 BC028528 Dmkn Foxo1 Nfkbid Cryab Padi2 Fam46c Chrdl1 Spic Dusp4 Vmn1r90 Ednra Galnt6 Abca8a Zbtb24 Cfd Maged2 Tagap Mefv Sox7 Ccbl2 Spn Fgd3 Icam4 Dnahc5 Rag2 Naip2 Cyp27a1 Ctsw Cacna1i Ryk Il2rb Cd93 Gpc3 Abcg1
**B**	241	Cldn1 I830012O16Rik Apobec3 Prkar2b Ntrk2 Tek H2-Q5 Myo5c Krt79 Camk2b Gucy1a2 Cyp2f2 Camp Igsf10 Mmrn1 Diap2 Cenpe Chia Gas2l3 Sh3d19 Olfml2b Crabp2 Pi16 Ms4a4c Scn3b Cd52 Dag1 Vasn Atp6v0d2 Siglece Mospd2 Cpxm1 Gpam Cpeb4 Nts Ar Fbln1 Tuba4a Nrip1 Dbp Thbd Cd247 Slco2b1 Abcc9 Acer2 Hey1 Sulf2 Rnf144a Nkx2-1 Serpina3g Clspn Prss22 Ccl22 Mertk Vps13c Slc9a4 Spag16 Tbl1x Ltf Niacr1 Mx2 Uty Slit3 Arid5a Ifit3 Ggcx Tnxb Adamts6 Cyyr1 AW549542 Gatad2b Dpyd Bmp5 Rgs5 Zbed6 Cnp Cckar Kdm5d P4ha1 Gzmm Bin2 Pygl Evi2a Glg1 Clec1a Hspb7 Ifi203 Aox3 Pign Notch4 Leprel1 Hspa12b Raph1 Wwp1 Hlf Itga1 Pydc3 Jag2 Ccdc149 Ccdc85a Fam181b Cxcr6 Ms4a6b AI839979 Rem2 Ifi27l2a Heg1 Mmp15 Rtp4 Tgtp1 Crybb3 Col28a1 AI506816 Rrad Nlrp1a C77370 Pyhin1 Il1rl2 Acaa1b Ccl6 Nrgn Ly75 Wisp1 Tgtp2 Eif2s3y Treml2 Ptprcap Mxi1 Foxn3 Ly86 Olfml2a Sfn Scn4a Cxcr4 Megf6 Psmg1 Gpr18 Fam168a Ereg Myo7a Hipk4 Lgi2 Zfp366 Plac9b Tmod2 Cd70 Mical3 Alas2 Sema3g BC094916 Cd248 Bag2 Itgb6 Slc25a36 Ccl2 Klf4 Col14a1 Vcam1 Dpys Caskin2 Ptgfrn Trim34a Tm4sf1 Wscd2 Pcdhb5 Mrc1 BC147527 Sema3d Scgb3a2 Cxcr1 Ston1 Trim2 Pim2 Col1a1 Pisd-ps3 Dmxl2 Ero1l Krt80 Serpinf1 Sell Asns Hivep3 Mmrn2 Casp4 Cped1 Lrp12 Usp18 Anpep Bpifb1 Foxo3 Tmem108 Sema3c Sytl4 Xist Glb1l2 Scara5 Itgbl1 Cxcl12 Fndc1 Mn1 Cebpa Xdh Scube1 Cars Per1 Dnmbp Cidec Tnfrsf18 Cd3g Nlrp3 Irx1 Tspan18 F3 Chst4 Ly6d Ngp Irf7 Gab2 Arhgap5 Slc25a22 Ms4a4b Slfn1 Cbl S100a4 Csprs Slc6a15 Psat1 Mx1 Gdf10 Nudt7 Elovl6 Cd79a Fzd6 Kcnj15 Tmem245 Daxx Tspan11 Arhgap19 Col15a1 Siglec1 Rora

Supplemental Table 2.

Screening of differentially expressed genes, up and down regulated, involved in oxidative stress and antioxidant defense response in the hydrogen peroxide pathways. The gene expression level is calculated by using RPKM method (Reads Per kb per Million reads). We used the False Discovery Rate (FDR)≤0.001 and the absolute value of log_2Ratio ≥1" as the threshold to judge the significance of gene expression difference. Genes have p-value< 0.05 and FDR≤0.001.

Gene ID	Symbol	log2 Ratio (Fold Expression)	Up-Down-Regulation	P-value	Name	Accession Number
**50490**	Nox4	1,52	Up	6,25E-26	NADPH oxidase 4	NM_001285835.1
**20657**	Sod3	1,39	Up	3,61E-130	superoxide dismutase 3	NM_011435.3
**13429**	Dnm1	1,38	Up	2,47E-06	Dynamin 1	NM_010065.3
**50997**	Mpp2	1,35	Up	1,26E-04	Membrane protein, palmitoylated 2	NM_016695.3
**50876**	Tmod2	0,91	Up	4,75E-11	Tropomodulin 2	NM_001038710.1
**67305**	Gpx7	0,90	Up	8,95E-04	Glutathione peroxidase 7	NM_024198.3
**21916**	Tmod1	0,73	Up	2,13E-02	Tropomodulin 1	NM_021883.2
**18127**	Nos3	0,54	Up	6,72E-04	Nitric oxide synthase 3	NM_008713.4
**16365**	Irg1	-0,74	Down	5,24E-292	Immunoresponsive gene 1	NM_008392.1

Supplemental Table 3.
Trp metabolites quantification by LC-HRMS.

Analyte	*Il1r1*$^{-/-}$ MEF cells (pmol/tot cells)	*Il1r1*$^{+/+}$ MEF cells (pmol/tot cells)
Kynurenine	24	14
3-hydroxyantranilic acid	<LOD	n.d.
Xanthurenic acid	<LOD	n.d.
Kynurenic acid	<LOD	n.d.
Quinolinic acid	<LOD	n.d.

The analysis has been carried out extracting the whole cell sample and performing a double LC injection; kynurenic acid, xanthurenic acid, quinolinic acid and 3-hydroxy-anthranilic acid were not detected (< LOD). n.d. not done

Supplemental Table 4.
Probe set information of the 50-plex QuantiGene assay.

Accession Number	Gene symbol	Probe Set Region
NM_009045	Rela	818-1198
NM_009994	Cyp1b1	1225-1752
NM_019911	Tdo2	2-631
NM_016666	Aip	72-449
NM_009709	Arnt	783-1237
NM_007818	Cyp3a11	6-457
NM_010699	Ldha	355-940
NM_007819	Cyp3a13	618-1071
NM_008683	Nedd8	18-523
NM_008324	Ido1	150-719
NM_008678	Ncoa2	435-857
NM_027552	Kynu	178-593
NM_011638	Tfrc	166-625
NM_013556	Hprt	207-860
NM_008361	Il1b	89-615
NM_007436	Aldh3a1	690-1101
NM_013464	Ahr	995-1543
NM_011424	Ncor2	1264-1707
NM_173391	Tph2	1160-1545
NM_009414	Tph1	554-1140
NM_001199212	Asmt	436-855
NM_008679	Ncoa3	2401-2820
NM_172778	Maob	261-860
NM_010936	Nr1i2	1179-1570
NM_145949	Ido2	46-481
NM_008360	Il18	81-577
NM_145827	Nlrp3	232-837
NM_177821	Ep300	6760-7134
NM_172404	Ccbl1	1036-1381
NM_011075	Abcb1b	22-669
NM_007956	Esr1	1215-1623
NM_008828	Pgk1	698-1301
NM_009029	Rb1	1945-2384
NM_009992	Cyp1a1	836-1409
NM_013467	Aldh1a1	1497-2011
NM_001174170	Serpinb2	367-811
NM_010480	Hsp90aa1	245-989
NM_009089	Polr2a	2581-3104
NM_019766	Ptges3	171-595
NM_173740	Maoa	1136-1563
NM_010145	Ephx1	582-1102
NM_009591	Aanat	823-1187
NM_133808	Kmo	267-617
NM_025325	Haao	487-851
NM_010881	Ncoa1	1367-1890
NM_172495	Ncoa7	2823-3303
NM_009644	Ahrr	654-1090
NM_016672	Ddc	189-628
NM_008181	Gsta1	229-710
NM_009460	Sumo1	24-455

Uncropped Figure 1G

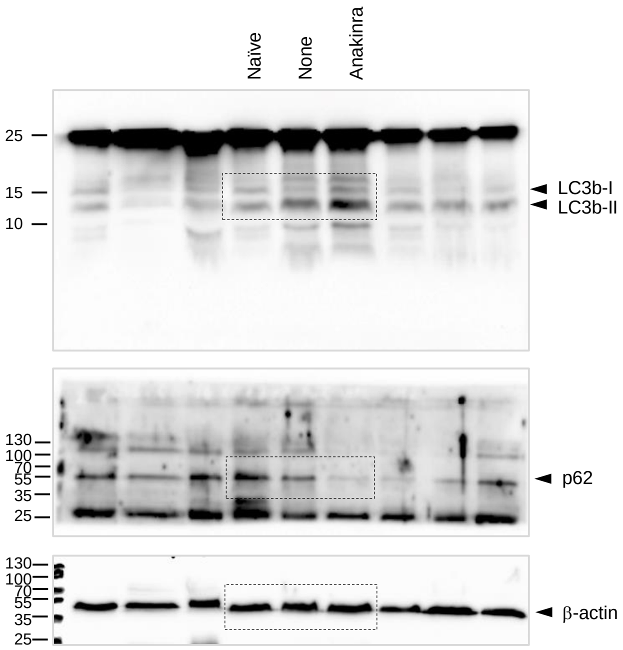

Uncropped Figure 3D

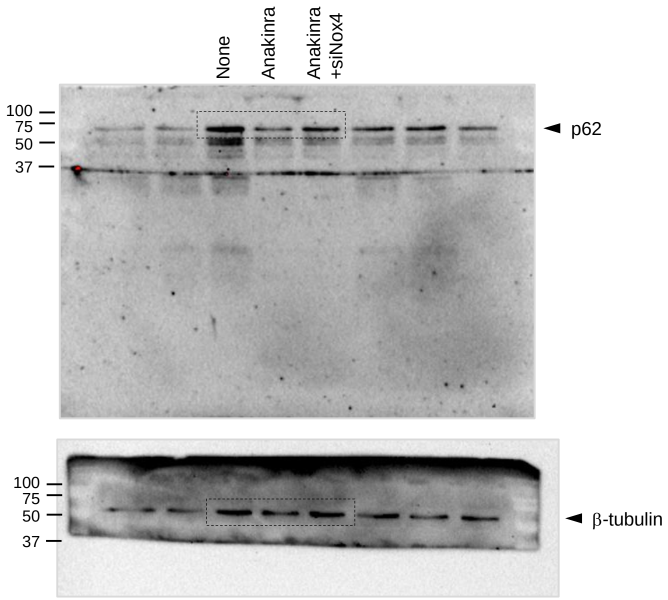

Uncropped Figure 4C

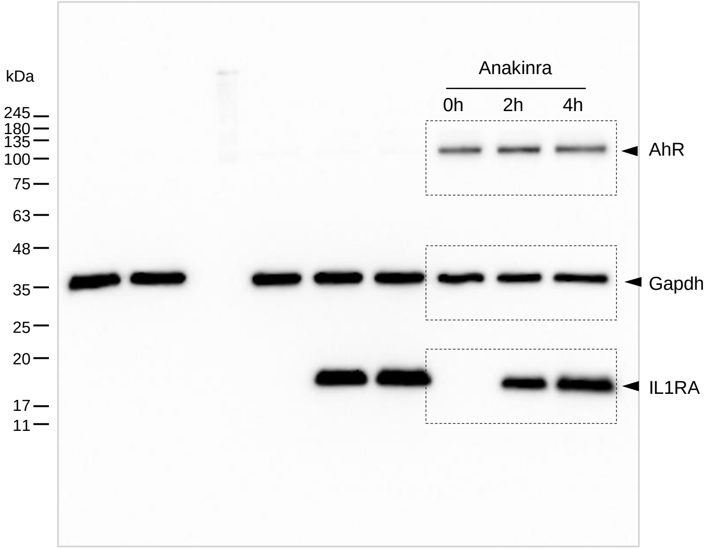

Uncropped Figure 4F

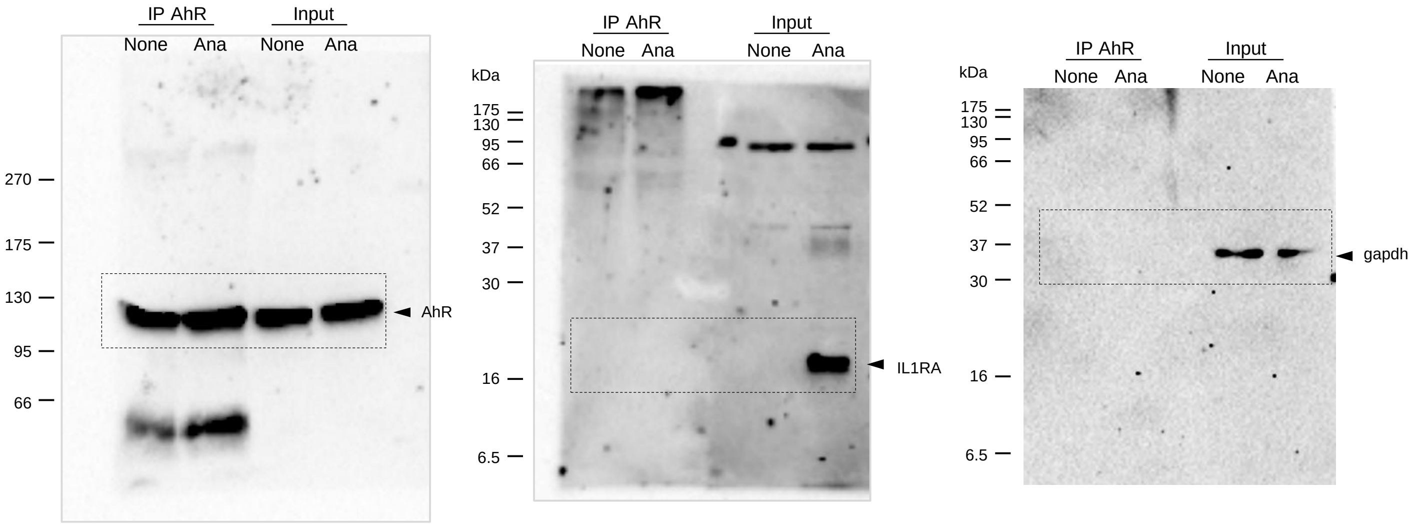

Uncropped Figure 5B

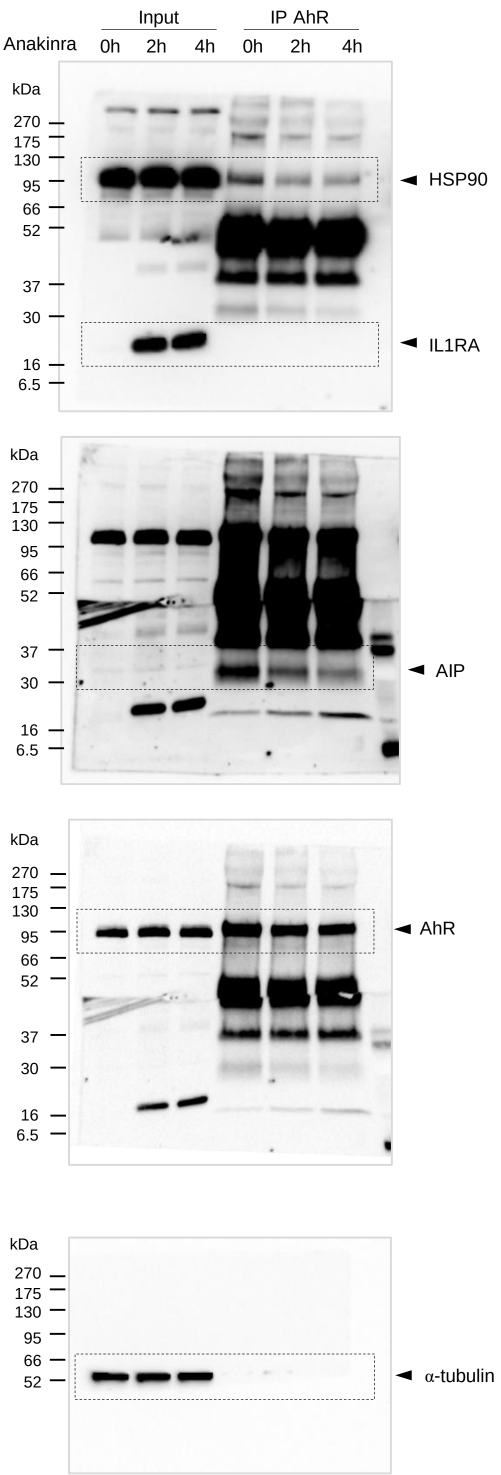

Uncropped Figure 5C

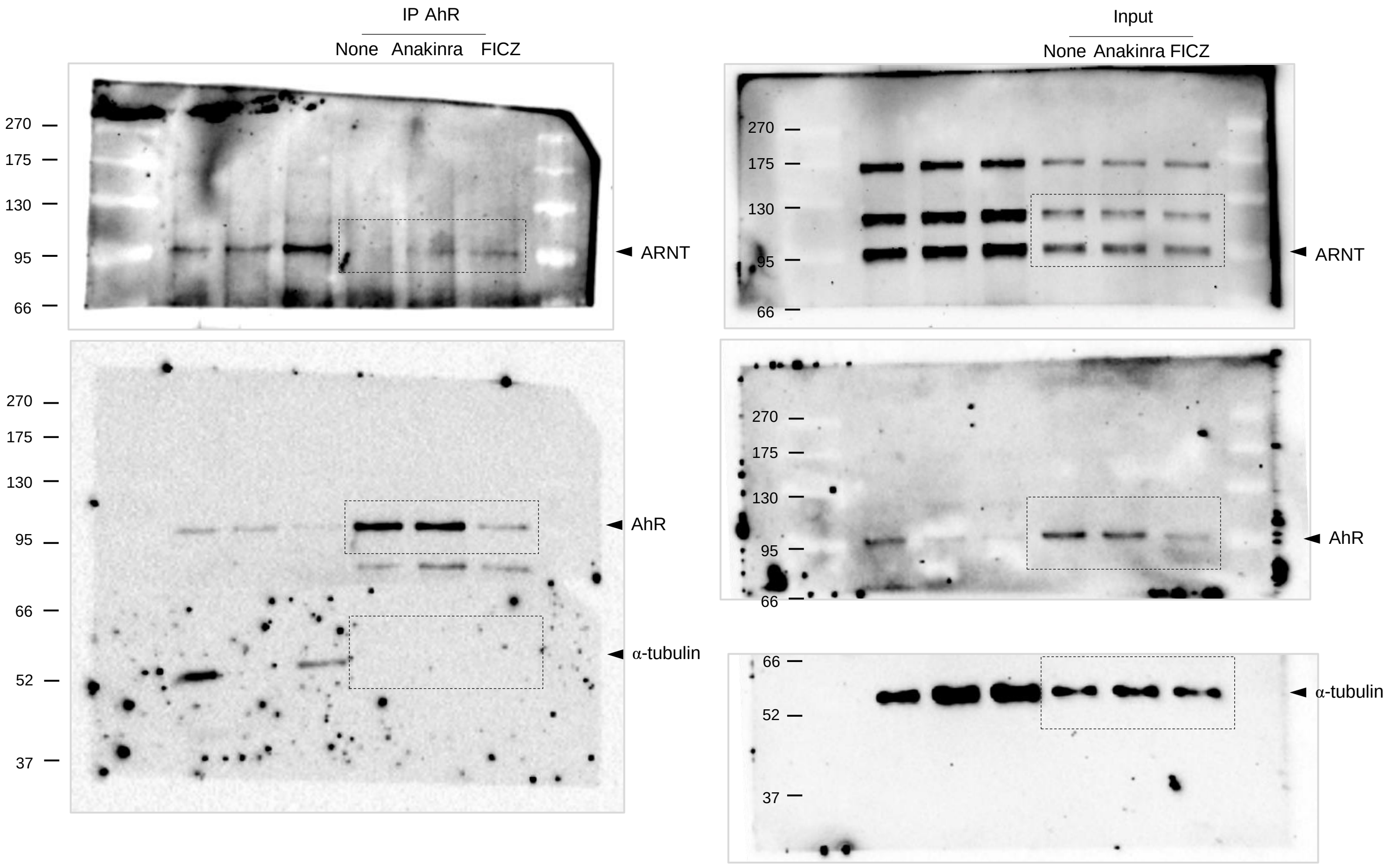

Uncropped Figure 5F

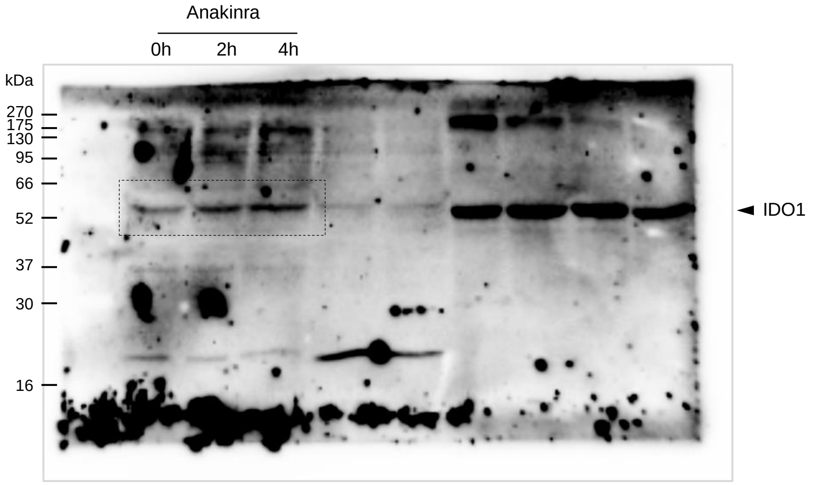

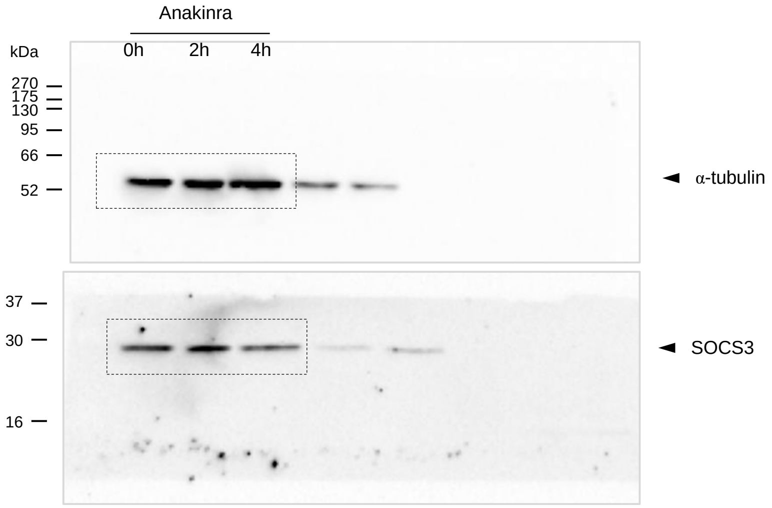

Uncropped Figure 6A

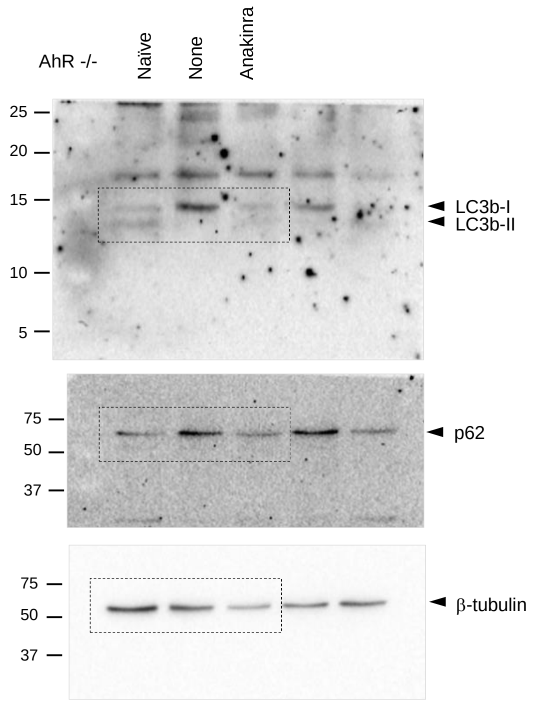

Uncropped Figure 8A

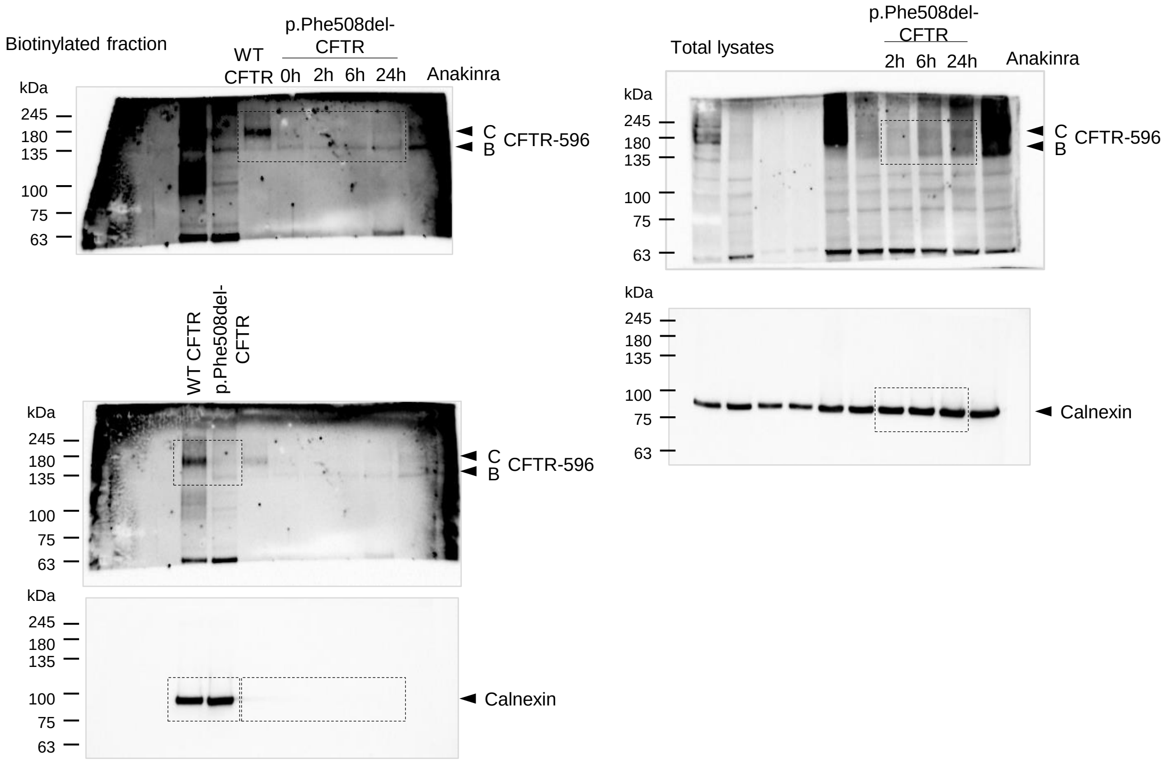

Uncropped Figure S3

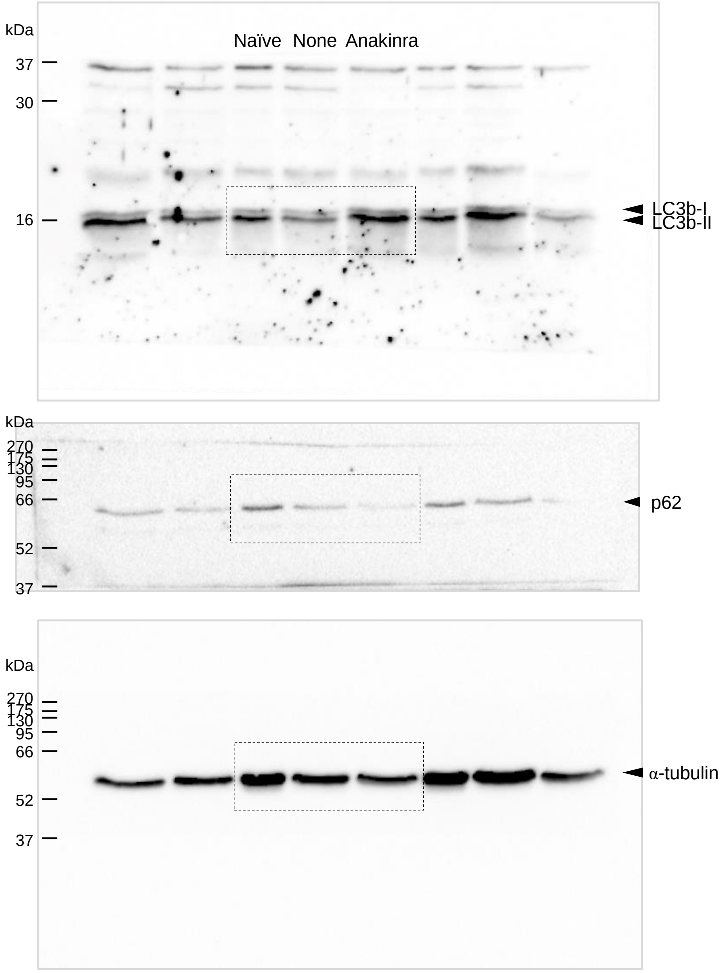

Uncropped Figure S9

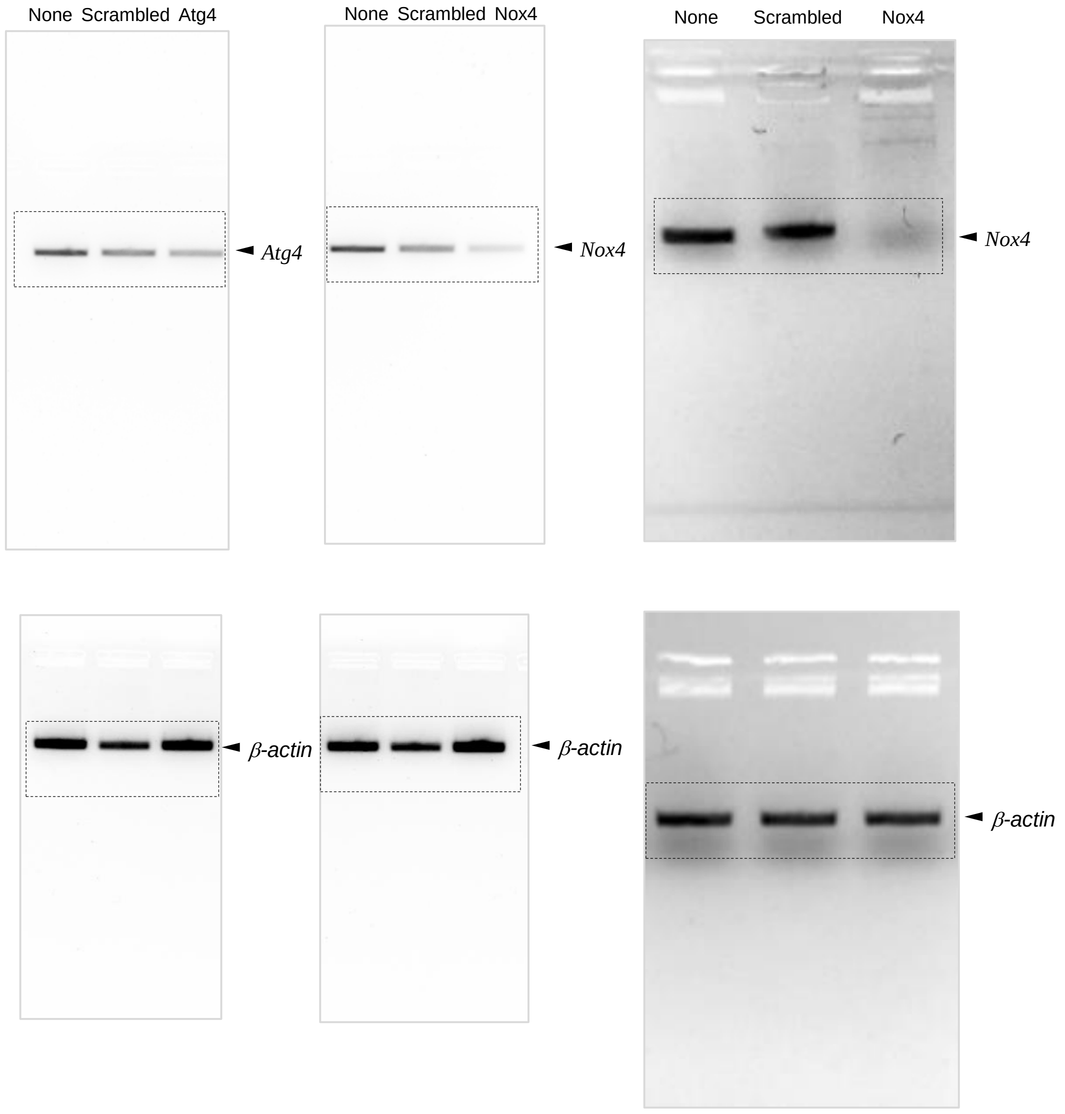

Uncropped Figure S11

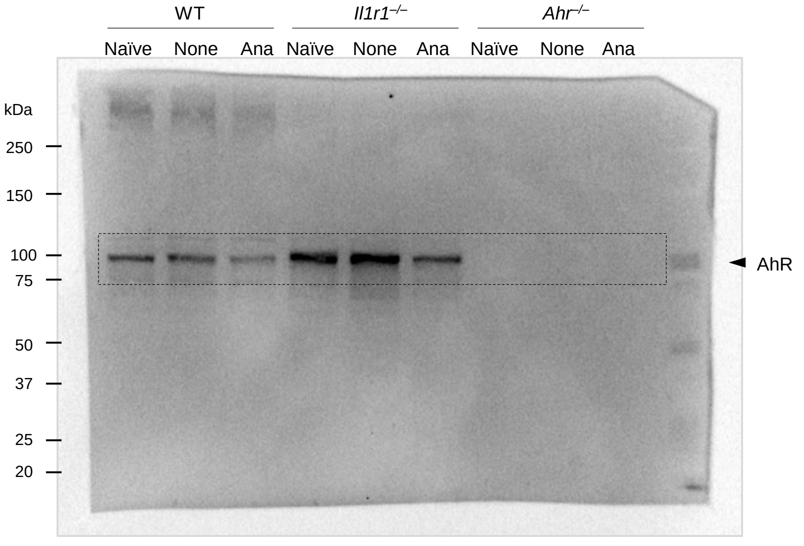

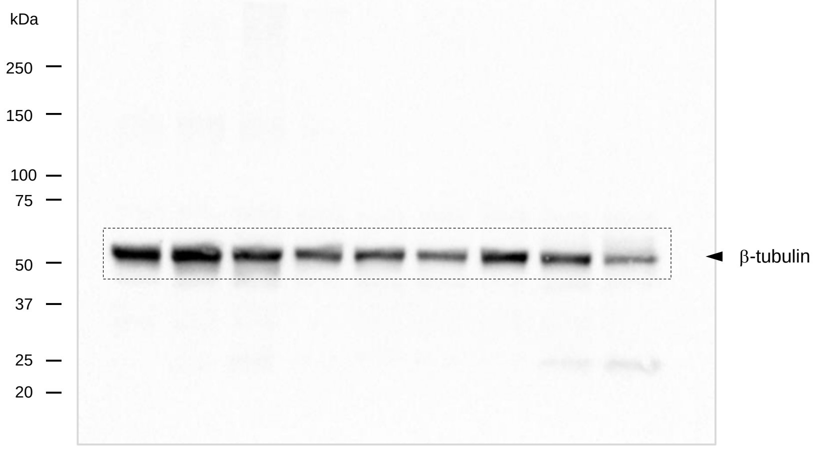

Supplement: Supplemental data [file jci-132-144983-s110.pdf]
